# Oleogels as a Fat Substitute in Food: A Current Review

**DOI:** 10.3390/gels9030180

**Published:** 2023-02-24

**Authors:** Roberta Claro da Silva, Md. Jannatul Ferdaus, Aline Foguel, Thais Lomonaco Teodoro da Silva

**Affiliations:** 1 Family and Consumer Sciences Department, College of Agriculture and Environmental Sciences (CAES), North Carolina A&T State University, Greensboro, NC 27411, USA; 2 Department of Biochemical-Pharmaceutical Technology, Faculty of Pharmaceutical Sciences, University of Sao Paulo, Sao Paulo 05508-000, Brazil; 3 Science des Aliments et Formulation, Gembloux Agro-Bio Tech, ULiège, 5030 Gembloux, Belgium

**Keywords:** oleogel, food, oleogelators, saturated fat, healthy

## Abstract

Fats and oils in food give them flavor and texture while promoting satiety. Despite the recommendation to consume predominantly unsaturated lipid sources, its liquid behavior at room temperature makes many industrial applications impossible. Oleogel is a relatively new technology applied as a total or partial replacement for conventional fats directly related to cardiovascular diseases (CVD) and inflammatory processes. Some of the complications in developing oleogels for the food industry are finding structuring agents Generally Recognized as Safe (GRAS), viable economically, and that do not compromise the oleogel palatability; thus, many studies have shown the different possibilities of applications of oleogel in food products. This review presents applied oleogels in foods and recent proposals to circumvent some disadvantages, as reaching consumer demand for healthier products using an easy-to-use and low-cost material can be intriguing for the food industry.

## 1. Introduction

The increase in the population’s demand and health institutions worldwide for healthier foods has been increasingly expressive due to the awareness and association of some foods with chronic diseases [1]. One of the leading causes of CVD, diabetes type-II, many metabolism disorders, and obesity is consuming foods with a high level of trans and saturated fats, which occur in many commercial products such as confectionery, bakery, lactic, and meat products. Replacing 5% of fat with polyunsaturated fats, like vegetable and marine oils, can reduce human CVD risk by up to 22–37% [2]. In May 2018, the World Health Organization (WHO) published a policy plan to eliminate trans fats, called REPLACE. The goal is that by 2023 industrial trans-fat should be eliminated from the food industry [3].

Trans and saturated fats provide technological and functional characteristics to foods like crispness, flavor, longer shelf life, and satiety that are challenging to replace. To substitute saturated and trans fats, food scientists explore lipid modification methods such as interesterification, fractionation, and blending [4]. However, none of these methods, until now, are satisfactorily reducing or eliminating trans and saturated fat levels without altering the product’s characteristics [5].

Fats such as palm and palm kernel have been used extensively and successfully in foods to replace partially hydrogenated fats. However, there is growing concern about using these fats, with their high saturated fatty acids (SFA) content and sustainability issues. Starch and natural gums (polysaccharides) are also studied and used to partially or entirely replace shortenings in confectionery products [6,7,8]. However, replacing saturated fats with refined carbohydrates does not give the same characteristics to the product. Additionally, it does not meet the current nutritional demands, as mono and disaccharide diets with low fat and highly refined content are associated with different health problems, including dyslipidemia, a component of the metabolic syndrome [9]. Therefore, searching the food industry for lipid sources that have the technical functionality and meet the new nutritional needs is necessary and unceasing.

Within this scenario, one of the most promising keys to replace saturated and trans fats, as shown in the studies selected in this review, is a method of structuration of lipids called oleogelation, which has been successfully applied to different formulations of oleogels in different food systems, or proposed solutions to overcome some limitations [10,11]. Oleogels are generally rich in unsaturated fatty acids (UFA) that acquire a semi-solid texture when trapped in a three-dimensional crystalline network formed by a structuring agent (oleogelator) at a specific concentration. Characteristics of each gel, such as viscosity, hardness, and melting point, can vary depending on the structuring agent, making it possible to develop different products [12]. In recent years, several structuring agents have been researched for edible oil structuring, such as vegetable waxes [13], monoglycerides [14], phytosterols [15], lecithin [16], and cellulose derivatives [17,18], among others. The combination of those gelators has also been explored [19,20,21,22,23]. The basic supra-molecular assemblies formed by these diverse molecules fall into one of the following predefined groups of oleogel structuration: (i) crystalline particles; (ii) self-assembled structures of low molecular weight compounds; (iii) self-assembled structures of polymers or polymeric strands; and (iv) miscellaneous structures like colloidal particles and emulsion droplets [24]. Due to the high structuration promoted by those different oleogel routes and the possibility to mimic high saturated fat behavior, many applications in food of these technologies have been performed on several food products, such as bakery [25,26], meat products [27,28], confectionary [29,30], margarine and spreads [31,32], ice cream [11,33], and others. Although several studies have been done, few were able to accomplish all requirements for a successful replacement: 100% replacement with good technological properties, sensory acceptable, and economically viable.

Moreover, there is no study of oleogels on a large scale. Thus, this review aims to give an overview of the potential of oleogel in different food products. First, it will discuss the different structuring techniques of edible oil with a broad range of oleogelators (oil structuring agents). Then it will focus on the available experiments of oleogels where they fulfilled the nutritional requirements, maintaining or improving the characteristics of traditional formulation products.

## 2. Lipids in the Food Industry

Food products that are sources of lipids are present in many categories, such as meat, bakery and confectionery products (bread, cakes, cookies, chocolate), dairy products (processed cheeses, ice cream), and spreadable products (margarine, fillings, toppings, mayonnaise). Lipids may also be commercially available as an ingredient, for example cooking oil, frying fats, vegetable creams, and animal fats [34].

Among lipids, fats are solid at room temperature; one example of a widely used fat in the food industry is shortening. Shortenings are commonly used in food industries due to their plasticity and physicochemical properties. Moreover, consistency and mouthfeel can even be tailored to the final product using different shortenings [35]. Initially, shortenings were made from partially hydrogenated fats containing large trans fatty acids (TFA). Then, as an alternative to decrease the trans portion of foods, other processes, such as total hydrogenation, interesterification, and mixing, began to be applied, massively replacing TFA with saturated ones [36].

Fats are mainly responsible for promoting specific attributes, such as structure, texture, creaminess, aeration stability, crispness, flavor, aroma, satiety, and oxidative stability required by each product [37]. The functionality of fats is even more diverse depending on the type, quantity, and food system in which they are applied. In bakery and baked goods, fats provide soft texture, a means of transferring heat during cooking, aid in flavor dissemination and permanence of flavor, incorporation, stabilization of smaller air bubbles, greater volume, more uniform porous structure, and prolong the shelf life of the product [38]. For ice cream, it is essential to have a smooth and soft texture, structure stabilization, and creamy mouthfeel [11]. For other dairy and spreadable products, fat imparts desirable flavor, mouthfeel, rheological properties, and softness and elasticity [39]. In meat products, saturated fat contributes to the texture, mouthfeel, juiciness, and consumer sensory acceptability [40]. In chocolate, desirable characteristics are a slightly crunchy texture when biting, brightness, and rapid melting in the mouth attributed to crystallization in the β polymorphic form, and its melting point (~34 °C) just below 37 °C, the average human body temperature [41]. Biscuit fillings, composed of an average of 40% fat, should be firm at room temperature, provide a smooth, creamy mouthfeel, and have no loss of oil or moisture. In addition, it must be firm enough to avoid misalignment and maintain its shape when handled or bitten [9]. 

As previously mentioned, even with all the functionality of trans and saturated fats, eliminating TFA and replacing SFA with mono and polyunsaturated fats (PUFA), primarily present in liquid oils, is recommended. Development efforts in the oil and fat industry are focused on reducing the SFA and improving foods’ nutritional characteristics. However, due to the desired characteristics for each type of product, the reduction of solid fat cannot be applied only by replacing it with liquid oil, as shown by several articles selected in this review, which experimentally compared the traditional product with a sample of the same product, of lipid phase replaced by oil [42]. An example is the fact that many sensory properties of food (spreadability, consistency, firmness) depend on the TAG crystal network; however, in the case of oleogels, there is some difficulty in predicting the type of structure of the structuring agents’ crystals and how they will behave in the different proportions and conditions in the oleogels [43].

## 3. Oil Structuring

**Figure 1:**
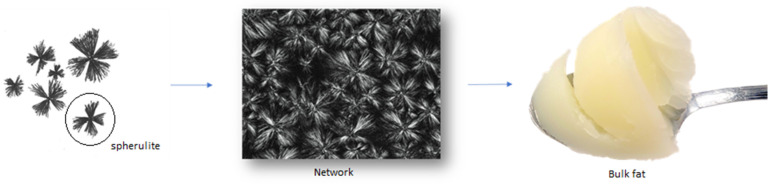
Illustration of a conventional fat crystallization. Attractive interactions between fat crystals form agglomerates, which interact and form a network.

An oil/fat is composed of a complex combination of triacylglycerols (TAGs). In fats subjected to temperatures above their melting point, nucleation events initiate due to the limited solubility of the molecules with the highest melting point, producing tiny crystals that grow and interrelate with each other through non-covalent forces, developing a continuous three-dimensional crystalline network (agglomerate). Finally, the crystals aggregate and form clusters, which constitute larger structures that form weak bonds. This leads to a macroscopic network, characterizing the conventional structuring of lipid systems based on TAGs [44]. The composition and distribution of the fatty acids along the glycerol chain and the crystallization behavior of these molecules (crystal morphology, polymorphism, etc.) are what define the behavior of fat as a semi-solid material [45]. Figure 1 illustrates the conventional crystallization of a fat.

**Figure 2:**
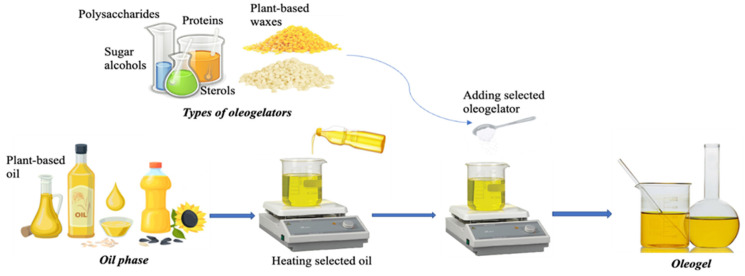
The general method of oleogel preparation.

The system called oleogel is considered an unconventional form of structuring lipids, as opposed to conventional structuring, in which the macroscopic properties of plastic fats, such as solid fat content, texture, and melting profile, are closely dependent on microscopic properties; in other words, dependent on the lipids’ crystalline microstructure. Therefore, oleogels generally comprise 90% liquid oil, and their properties are similar to solid fats. However, in unconventional structuring, instead of only TAGs, the three-dimensional network is formed by an oleogelator (structuring agent), whose ability to gel an oil depends on an equilibrium between the solubility and the insolubility of this agent in the oil; that is, it must be relatively insoluble to crystallize or self-assemble, while it must be relatively soluble to interact with the oil molecules [46]. Figure 2 shows a general method for oleogel production.

Oils represent the most significant fraction of the oleogel, on average 90% of the composition. The oil must have availability, functionality, low cost, economic importance, and be nutritionally beneficial for industrial applications. The main oils used in the latest studies have vegetable origins, like soybean, olive, sunflower, and high oleic sunflower oils [47]. Puscas et al. [34] reported that the minimum structuring agent concentration is affected by saturation levels of oils. Higher proportions of SFA resulted in the need for less structuring agent in oleogelation.

**Figure 3:**
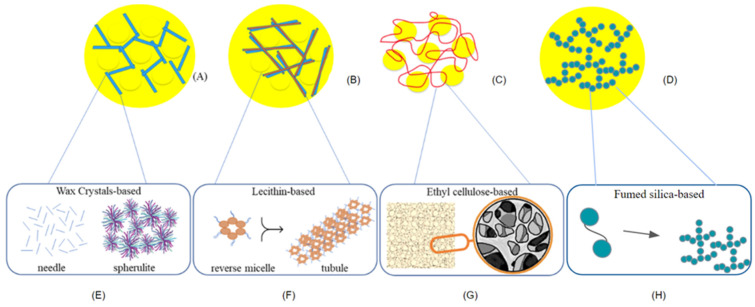
Oily continuous phase. Classification of oleogel formation: (**A**) Crystalline particles; (**B**) Self-assembly; (**C**) Polymeric agent (hydroxyethyl cellulose); (**D**) Inorganic particles (fumed silica); (**E**) Crystal structure example (vegetable wax); (**F**) Example of self-assembly structure (lecithin); (**G**) Example of structure with hydrophobic polymers (ethylcellulose); (**H**) Example of organized structure with inorganic particles of fumed silica (interaction between two colloidal silica particles (**Left**) and the formation of chain structure (**Right**).

According to the mechanism associated with the structuring of TAGs, oleogelators can be classified into two groups—the conventional approach using crystalline TAGs as building blocks (Figure 3A), and the alternative approach using non-TAG building blocks, which are formed by self-assembled structures (Figure 3B), polymeric strands (Figure 3C), and inorganic particles (Figure 3D) [24].

Crystalline molecules have been associated with the classic phenomena of nucleation, growth, and stability of the crystalline network [48]. Oleogels of crystalline particles are usually formed by crystalline particles like natural waxes, monoglycerides (MAGs), diglycerides (DAGs), fatty acids, fatty alcohols, cholesterol, D-limonene, long-chain triglycerides, dicarboxylic acids, phytosterols, and sorbitan esters [49]. Most crystals in lipids have three polymorphic structures: α, β′, and β. The α form is metastable and of hexagonal chain arrangement. The β′ form presents intermediate stability and perpendicular orthorhombic arrangement, while the β form presents greater stability and parallels the triclinic arrangement. The stability grows due to differences in the molecular arrangement density as the melting temperature increases [50]. Crystals in the β′ form show better functionality, as they are smoother and guarantee good aeration and creaminess attributes. Then, the polymorph β′ form is interesting for producing food products such as margarine and icing due to the emulsified lipid phase [51]. Some oleogelators, such as beeswax (BEW), can form this type of β′-crystal similar in shape to needles, platelets, and spherulites, to β′-crystals formed by fats depending on their concentration and the oil used. The concentration of this structuring agent also interferes with the crystal network. The higher the concentration, the more compact the network can be. The high degree of supersaturation accelerates nucleation and gelation [52].

The oil structuring by the self-assembly systems is facilitated by a mechanism of molecular self-organization of its components in the organic phase [9,24]. Low molecular weight compounds can form this structure, such as ricinelaidic acid, hydroxy stearic acid, phytosterols + sterol esters, lecithin + sorbitan tristearate, sphingolipids, phospholipids + tocopherols, and others. Alternatively, high molecular ones, such as polymers and proteins, can be hydrophobic such as ethylcellulose (EC), or hydrophilic such as methyl cellulose (MC), hydroxypropyl methylcellulose (HPMC), and proteins [49].

Hydrogen bonds, electrostatic interactions, and Van der Waals covalent are examples of intermolecular interaction forces of the oleogelators that form the three-dimensional gel network by self-assembling low molecular weight compounds [7]. Component parts with similar molecular and chemical structures often show synergic interactions about dispersed particle phenomena and self-association. Particle self-assembly can occur in non-crystalline particles such as tubules, fibers, or reverse micelles, as shown in Figure 3B.

Self-organization of polymers in the oil phase occurs with the formation of polymeric fibers and, thus, gelation (Figure 3C). Gelation can occur in chemical gels through covalent bonds and in physical gels through self-assembly via hydrogen (such as EC) or van der Waals forces [7]. For instance, during the gelation of the oleogel structured by EC, the structure presents a network of voids, which suggests that the oil is compartmentalized in distinct pockets (Figure 3C). Gravelle and Marangoni’s analyses [53] show that a network of capillary channels in this system probably connects oil droplets. If a structuring agent is very soluble in oil, a solution is formed instead of a gel, and if it is very insoluble, it will not interact with the oil and form sediment instead [54]. When the hydrophilic options are used as oleogelators, they demand indirect approaches, as the oil is unable to interact directly with the polysaccharide. The two indirect procedures are known as foam template (dissolving the gelator in water followed by freezer-dryer) and emulsion template (forming an emulsion and subsequently evaporating the water); after this method, the material formed is further dissolved in oil [55].

The inorganic particles form into three-dimensional anisotropic networks, which have comparable structural and mechanical characteristics to networks of fat colloids (Figure 3D). For example, the dispersion of fumed silica in a TAG solvent at a high concentration (10 wt% and above) results in the formation of a gel where the liquid solvent is physically trapped in a 3D network based on the fractal aggregation of silica particles [56]. Inorganic structured oleogels could improve the nutritional quality of edible oleogels by modulating the lipid digestion rate and releasing active ingredients encapsulated within the networks [57]. The results of the various studies included in this review show that methods/processes of forming oleogels, the types and fractions of oils, and the structuring agents used will micro and macroscopically influence all the oleogels formed. Consequently, the properties of the final food product depend on these same factors, plus the interference of the other ingredients in the formulation.

## 4. Application of Oleogel in the Food Industry

To lower SFA in the diet, the structuring, characterization, and application of oleogels have been receiving attention from researchers and the food industry [38]. Another advantage is that according to the application, the structural and physical properties of oleogels can be engineered by different factors, such as the type of vegetable oil, concentration of structuring agents, and processing conditions. Furthermore, the types of structuring agents and oil are fundamental to determining the crystalline structures and physical properties of oleogels [24,58].

In addition, GRAS-status oleogels can act as dietary supplements, offering an alternative for applications that rely on releasing hydrophobic bioactives in living tissue [59]. Furthermore, oleogels may be responsible for increasing the bioavailability of some active substances, such as lycopene, phytosterols, and vitamin E, thus reinforcing yet another future application in food [60].

There are still only a few studies on the digestibility of oleogels; however, Tan and collaborators [61] published the first human study on the induced decrease in oil bioaccessibility (a macronutrient) due to gelation with EC. The postprandial decrease in serum TAG levels is associated with a risk reduction of CVD. Limpimwong and collaborators [62] investigated the effects of the structure of the rice bran wax (RBW) network in oleogel during lipid digestion in rats compared to the consumption of margarine and bovine tallow. Rats fed a high-fat diet for four weeks on oleogel reduced the TAG level (30%) in the serum and liver, the growth of adipose tissue, and total cholesterol in the liver. In addition, there were increased excreted levels of TAG (30%), total cholesterol, and bile acid in the feces compared to tallow and margarine. It was concluded that the gel network significantly contributes to the decrease in lipid digestibility.

Additionally, Dong et al. [63] examined how self-assembled crystalline structures affected the ability of phytosterol oleogels to be digested. Results showed that lipid digestion, including its emulsification effectiveness and additional lipolysis, was significantly influenced by the gel strength and the crystal nature. The gel crystal’s shape progressively altered with fluctuating phytosterol molar ratios, eventually impacting its physical characteristics. The quantity of surface area generated during the emulsification process significantly determined the degree of lipid digestion. According to the isothermal titration calorimetry findings, the oleogels’ microstructures acted as barriers that prevented lipase and the lipid substrate from interacting. Consequently, oleogel emulsions had less lipid digestion than oil emulsions. It confirmed the advantages, from a dietary point of view, of the different types of oleogels in food applications.

Even though most studies on oleogels are focused on analyzing the physicochemical aspects, some studies address applications in food systems, and the success of their work results in patents. Around 50 patents have been registered in the last ten years, demonstrating the potential of these applications in the industry [64].

Through the selected studies, some points were observed:

i. Products already well known and produced by the industry were chosen, rich in trans and/or saturated fat, in which fat plays an essential role in the physical and sensory characteristics of the product.
ii. Mostly, replacing traditional fats with oleogels is a priority to enhance the nutritional quality of the product. Other goals include stabilizing fat or aeration, delaying oxidation, or reducing product cost.
iii. Analyses such as rheology, microstructure, color, and texture show the concern for understanding the structure of oleogels within a food system, compared with the traditional product.
iv. Some more recent studies also include sensory analysis, demonstrating scientists’ awareness of the importance of consumer acceptance in developing a product.

**Figure 4:**
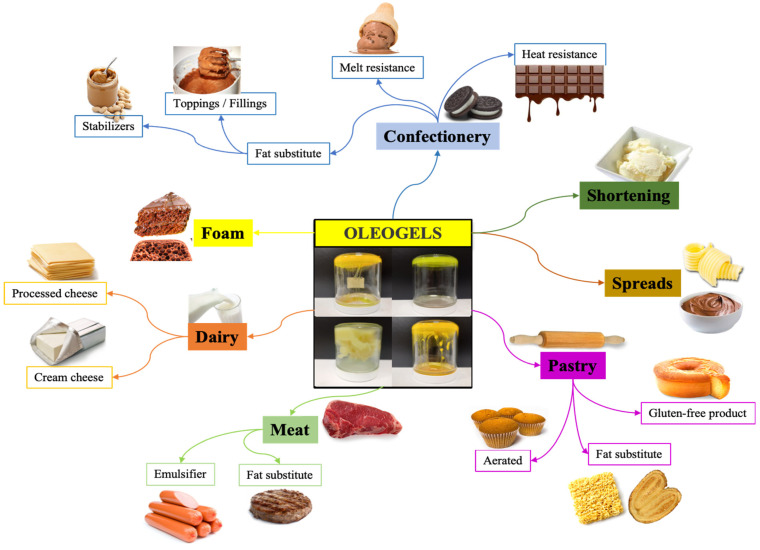
Application of oleogels in different food products.

The following topics present the selected studies, divided by categories of food products where oleogels were applied. Some application examples are presented in Figure 4 and will be described below.

### 4.1. Baked Products

The fats used in baked products play an important role in incorporating and stabilizing air for homogeneous growth. The creamy texture and solids content maintain the dough’s structure and viscosity. Oleogel typically presents a lower viscosity than shortening, which can interfere with air incorporation, decrease firmness, and increase mass density [65]. The most studied products are variations of biscuits/cookies and cakes/muffins due to the higher fat content of those baked products. A summary of the applications discussed on this topic is shown in Table 1.

#### 4.1.1. Cookies

Oleogels structured by wax were the most evaluated for shortening replacement in cookies. The studies, in general, have evaluated the different types of waxes in different concentrations using different oils. Jang et al. [66] used candelilla wax (CLW) in canola oil (3 or 6%). A lower hardness of the CLW oleogels compared to the shortening resulted in cookie dough with lower viscoelasticity. The oleogels were substituted for shortening to generate cookies with soft biting properties, and their decreased viscosity at baking temperature gave the cookies ideal spreadable features (lower snapping force). Additionally, compared to the shortening used in cookies (47.2%), the amount of UFA in the oleogel cookies was much higher, up to almost 92%.

Mert and Demirkesen [67,68] found the same results as Jang et al. [66] for CLW and carnauba wax (CRW) in sunflower oil (2.5 and 5%, respectively). Texture measurement showed that CRW and CLW oleogels could not make the short dough like regular shortening. While they discovered a significant improvement in the oleogel-prepared cookies compared to those made with sunflower oil alone, they also confirmed the disadvantage of using just liquid oil [68]. Further, they discovered that oleogel contributed substantially greater extensibility and lower hardness to the samples than shortening, which was explained by the wax-oleogels’ shear sensitivity when the CLW oleogel was created in a shear pilot scale [67]. For this reason, oleogel again was not able to produce similar cookies as shortening when the shortening was fully replaced. Nevertheless, when using CLW oleogel in blends with shortening, the technical characteristics of the cookies, such as hardness, spread ratios, and appearance, were unaffected when up to 70% of the shortening was replaced with 6% CLW oleogel, or 40% shortening with 3% CLW oleogel. These results suggested that using oleogels to substitute shortening in certain amounts, rather than all of it, may produce considerably more desirable dough and cookie qualities [67]. Recently, Pang et al. [25] found the same level of replacement for RBW (6%) oleogel, 7:3 (oleogel:shortening) in shortening. The oleogel-cookie exhibited a similar hardness, crispness, color, and sensory score compared to a 0:1 shortening cookie. Authors have also evaluated BEW, candle wax (CDW), and CRW; BEW and RBW oleogel were the most promising replacements [25].

To develop cookies with three distinct liquid phases, including OO, SBO, and FXO (8% wax-oleogel), Hwang et al. [69] investigated the potential of natural waxes (SFW, BEW, and RBW) as structuring agents. With a focus on the toughness of the SFW-FXO oleogel, both the individual waxes and oils considerably changed the oleogel attributes, including hardness and melting tendency. Similar to those for oleogels, dough attributes like toughness and melting characteristics were also significantly impacted. SFW and RBW gave dough the most firmness. Nevertheless, using waxes and oils did not substantially modify the firmness and spread factor of cookies containing oleogels. Cookies created with wax-OO oleogels had characteristics like those made with conventional margarine [69]. In this study, before the production of the cookies, the oleogels were converted to margarine (20% water emulsions) and also used a higher concentration of wax, which might be made possible by the total replacement of the lipid phase by the oleogel. The improvement of the physical properties of oleogels by adding small amounts of water was previously stated [81].

Although oleogels performed well as a partial replacement for shortening in cookies, no research has attempted to establish a link between the crystal structure and physical characteristics of oleogels and the overall sensory and textural qualities. Maybe because of this, there was only a partial replacement. Based on that, Li et al. [70] determined the typical indices for assessing the quality of innovative cookies created using wax oleogels, and the impact of the structural and physicochemical properties of oleogels on the quality of cookies. As a result, SFC, β’-crystals, and elastic modulus (G’) were discovered to be significant parameters that controlled the desirable qualities and attributes of cookies made using wax oleogels. Furthermore, they claimed that RBW and CLW oleogels created soft cookies with the proper spread and color combined with a 5–9% oleogelator. Moreover, oleogels with lower SFC (4–11%), higher amounts of β’-crystals, and higher density of crystals formed cookies as hard as with shortening. Moreover, the final hardness of the cookie was not correlated with crystal size [70].

Besides waxes, recently, others structuring agents have been evaluated in oleogels to produce cookies. Li et al. [71] evaluated BEW, RBW, HPMC, MAG, and sodium stearyl lactate (SSL) in 6% oleogels. MAG and RBW oleogel cookies showed excellent properties, and they were similar in texture, microstructure, color, rheological properties, and sensory acceptance as the shortening cookie. The melting qualities of the structural agents, mainly due to the baking process, are what the authors feel to be the key to whether they were successful. When the bubbles bake, the residual crystal-water interface acts as a second interface layer, allowing the bubbles to expand without breaking, melting the fat crystals and transferring them to the inside of the bubbles. Possibly, cookies made with HPMC were more challenging due to the HPMC oleogel’s inability to melt even at temperatures of 80 °C. When heated, MAG and RBW-based oleogels showed a broad range of melting behavior and strong viscoelasticity, whereas SSL had a narrower melting range and lower viscoelastic properties [71]. Authors have also seen some potential in SSL (emulsifier). They further evaluated different emulsifiers as gelators to replace shortening in cookies. MAG, SSL, PGE, and SPAN 60 at different concentrations (3, 6, 9, 12, and 15 wt% for MAG and SSL; 9, 12, 15, and 18 wt% for PGE and SPAN) in corn oil have been tested. At concentrations between 6 and 15% and 12 and 18%, MAG and SPAN cookies, respectively, exhibit shortening-like firmness and color, and their cross sections were consistently porous. Cookie toughness was unaffected significantly by the SFC and α-crystal of oleogels. In addition, oleogel cookies produced with emulsifier gelators with higher hydrophile-lipophile balance HLB values had decreased toughness. The important element for softer cookies in these data was determined to be the greater shear viscosity of emulsifier-based oleogels at 25 °C [72]. Findings contradicted the notion that SFC and β′-crystal are crucial factors in predicting and assessing the quality of conventional oleogel cookies. This work demonstrated a novel strategy for directing the quality assurance of cookies made using emulsifier-based oleogels [72].

In order to find full replacement alternatives for cookies, recent studies have also explored combinations of different oleogelators. After evaluating different combinations of SFW, MAG, and EC in rapeseed oil, three oleogels, SFW10, SFW5EC5, and EC5MG5, were used for cookie preparations after evaluation of plasticity, malleability, suppleness, and dough-forming characteristic of these different oleogels. Cookies produced with those oleogels were softer, a little brittle, fissured, and with lower height. Moreover, the oleogels were not as sensory acceptable as the traditional cookie, suggesting further evaluation and more studies for this kind of system [73].

Conversely, combinations of β-sitosterol and MAG oleogels (10% oleogels, 1:1) were also tested. In this instance, cookie properties prepared with the oleogels were compared with those made with market-available shortening. In addition, the authors evaluated raw and refined SBO as the oil phase. Concerning weight, thickness, spread ratio, and cookie hardness, it was found that refined and raw oil performed as well or even better. Physical characterization and cookie-making performance data may be used to conclude that unrefined and refined soybean oleogels may mimic conventional shortening, opening the door to the potential of employing oleogels in place of shortening in the baking sector [74]. MAG (0–10%) was also used to reinforce the 1.5% HPMC foam-structured oleogel, forming a double network lipid-based oleogel for partial replacement (50%) of the commercial butter in cookies. MAG concentration in the soybean oil significantly improved the hardness, fracturability, and chewiness, while cookie cohesiveness, springiness, and gumminess decreased significantly [75]. 

In summary, according to these studies, RBW, MAG, and MAG:β-sitosterol are good alternatives when the goal is to replace shortening in cookies using fully lipid oleogels. Nevertheless, waxes such as BEW, CLW, and SFW are alternatives if structured as water in oil oleogel-emulsions.

#### 4.1.2. Cakes

Sponge cakes and muffins were the most evaluated products. Muffin batters with reduced viscosity and shear-thinning activity were created when muffins were developed with 4% HPMC foam oleogels [76]. Viscoelastic studies revealed that HPMC oleogels contributed more to the viscous character of muffin batters. However, the specific gravity of cake batter tended to rise when replacement quantities of shortening were increased. Up to 50% of shortening substitution did not substantially affect the particular volume of cooked muffins. Moreover, the air cells in muffins made with HPMC oleogels were substantially bigger and were not evenly distributed. The HPMC oleogel substitution for shortening up to 50% by weight did not negatively impact the muffin’s soft and chewy texture [76].

Furthermore, muffins have been produced with MAG oleogel from HOSO [26,77]. In the formulation of muffins, conventional margarine was swapped out for the optimized oleogels, resulting in items with improved spreadability, increased specific volume, comparable toughness values, and a more linked and uniform crumb structure. After 10 days of storage, these items showed a lower oil migration of almost 50% compared to the standard muffins, demonstrating that the optimized oleogels (~6.6% MAG) may be utilized successfully to lessen oil loss in this delicious baked good [26]. A thorough sensory analysis was also carried out; flavor and sponginess were comparable between the reference and oleogel-based muffin cakes, and the former received better marks for appearance, color, and overall quality, showing that customers preferred the latter. In addition, the reformulated muffins exhibited a considerably better lipid profile, with a 68% decrease in SFA and an almost four times increase in monounsaturated fats. Our findings suggest that muffins made with oleogel might be suitable for introducing customers to a new product with a healthy fatty acid composition [77].

A combination of the two described systems, HPMC and MAG, named double network oleogels, was used to prepare cakes to replace 50% of the commercial butter. Baked cakes showed that enhancing the MAG crystal network also significantly improved the hardness and chewiness of cakes with HPMC [75].

Sponge cakes produced with partial/total butter replacement by 5% CLW:canola oil oleogel were evaluated (0, 25, 50, 75, 100%). In this research, the impact of oleogels on the digestion of cake starch, as well as their sustainability as replacements, were also assessed. While the viscoelasticity and toughness of the cakes were decreased with higher replacement percentages, the specific volume rose from 1.91 to 1.98 cm^3^/g. The decrease of amorphous domains was associated with creating hydrated and simple crystalline starch structures, with the short-range ordering rising by roughly 120%, according to Fourier transform infrared (FTIR) research. The in vitro starch digestion was improved by oleogel inclusion, which increased the digestible starch fraction from 70% to roughly 84%. Overall, the findings indicated that when using oleogel to make sponge cake, there is a trade-off between enhancing the textural qualities and boosting starch digestibility [78].

In sponge cakes, SFW, BEW, RBW, and SFW:BEW-based oleogels and oleofoams in canola oil were substituted for palm oil. Unfortunately, not all of the benefits of oleofoams could be transferred to the cake samples. In terms of oil-leaping and aesthetics (volume), all oleogels outperformed the canola oil type. The best oleogel for oil immobilization was the SFW:BEW combination, which produced migration rates that fell in the middle of those of canola oil and palm oil. Sensory studies showed few differences between the various sponge cakes, except for the overall preference, where the oleogel-based sponge cakes outperformed those made with canola or palm oil [43].

A complicated oleogel system was also assessed for use in cakes. In sponge cakes, a system made of whey protein, MAG-DAG, and BEW was investigated as a complete substitute for butter [10]. Including an emulsifier and whey protein in the oleogels fixed the aeration issue, which was previously detected in the same type of sample [82]. Statistics showed that the increased concentrations of oleogel and whey protein resulted in decreased toughness of the sponge cake. Moreover, whey proteins increased the batter’s aeration and sample porosity. The mixture optimization showed that 76.98% OO-BEW oleogel, 7.28% MAG-DAG, and 15.73% whey protein is the optimal combination to replace the butter in sponge cake properly. In addition, the cake’s shelf life was unaffected by this substitution [10].

According to these results for cakes, the complete replacement of the shortening from a technological and sensorial perspective was successfully achieved by using MAG or combinations of MAG-DAG with BEW. Although SFW:BEW also showed a satisfactory result, the product was not similar to a commercial control one.

#### 4.1.3. Other Baked Products

MAG or RBW at a concentration of 10 wt% in high oleic soybean oil were applied to bread and cracker formulas as a shortening replacement. Rapid viscoanalysis implied that MAG oleogel yielded a longer peak time and higher pasting temperature, indicating the potential to delay the gelatinization of the bread and cracker doughs. The measurements of farinography and rheology suggested that the shortening replacement with MAG and RBX oleogels did not significantly change the dough’s properties. Regarding the baked bread, all fats, including the oleogels, showed good symmetry and shape, with small crumb cells, and the crust color was uniformly distributed for all the bread types. Only MAG oleogel exhibited a softer crumb firmness. In crackers, the replacement with RBX and MAG oleogels did not substantially affect the viscoelastic behavior of cracker doughs. However, hardness was again reduced by MAG oleogel. Fat with a higher solid fat content tends to produce a harder dough, as in shortening. Since RBX and MAG oleogels contained the same oil, the difference in the cracker dough hardness might be caused by the properties of the gelators [79].

The process dynamics of oleogel-based tender dough products structured by CRW, β-sitosterol:BW, β-sitosterol:lecithin, and MAG were evaluated. Regarding the dough, the sample prepared using oleogel with CRW showed the strongest hardness (92.49 N) compared to the reference (21.80 N). All the oleogel-containing doughs had elastic solid-like behavior. The biscuits formulated with commercial margarine registered a hardness of 28.74 N. Samples with oleogels showed a specific tenderness for tender dough products, thus being suitable for this type of product (11.22–20.97 N), especially for the samples structured with β-sitosterol mixtures and CRW [81].

### 4.2. Dairy Products

Some applications of oleogels on dairy products are summarized in Table 2.

#### 4.2.1. Ice Cream

One of the most desired characteristics of this product is that it does not lose its structure quickly during consumption, with the freezer temperature increasing to room temperature. Ice cream is a complex product whose composition needs to contain a minimal amount of crystalline fat, which enables the development of structure-forming aggregates by partial coalescence, preventing the simple substitution by a liquid oil. Incorporating air and protein also forms ice cream’s traditional structure [33].

Zulim-Botega and collaborators [11,83] tested the functionality of oleogels in ice cream through variations on the formulation of oleogels made of HOSO, CLW, CRW, and RBW (10% wax content) as the oleogelators, and MAG or Polmo as an emulsifier. In the first study [11], the authors investigated the application of oleogel made with HOSO and RBW. The results showed that higher amounts of the Polmo emulsifier (80% mono and diglycerides and 20% polysorbate 80) reduced the adsorption of proteins by the oleogel droplets and increased the fat aggregate at the interface with air bubbles. The oleogel ice cream air bubbles were smaller than the control sample made with HOSO without a structuring agent, and the fat destabilization was similar to the control sample made with milk fat. However, despite the amount of Polmo, the structural collapse rate during ice cream melting with RBW was similar to the HOSO control and much faster than the milk fat control sample. This suggests that, although the structure of the oleogel had characteristics similar to those of milk fat, it did not build a structure capable of retaining its shape during melting. Compared with ice cream made with HOSO without a structuring agent, the oleogel provided a sample with a better texture and appearance. It was suggested to investigate the factors that contribute to the structuring of the oleogel. In the second study, the group evaluated the best structuring agent among RBW, CLW, and CRW waxes, the influence of different emulsifiers, the best concentration of the lipid phase, and the best ice cream processing conditions. Among the results, RBW gave oleogel a more remarkable ability to form and sustain the ice cream structure. The possible justification is that the other waxes have more complex compositions that interfere with the structure of the oleogel. MAG provided more stability than Polmo to the oleogel ice cream due to the better interaction between the crystallization of the fat and protein, but was still not equal to the interaction of milk fat with protein, even without the emulsifier. The continuous freezer process showed greater structural integrity than the batch freezer, even with larger fat globules, which would supposedly result in less stability. With a lipid fraction of 15% instead of 10%, the oleogel ice cream showed greater stability in the system, approaching the melting behavior of the control made with milk fat. This second study put solutions to the challenges found in the first, concluding that with formulation and manufacturing process modifications, it was possible to create an ice cream with a melt-resistant structure under ideal conditions [83].

A third study, focused on ice cream fat reduction using wax-oleogel, was recently performed using CRW wax [84]. Authors have studied the physical and sensory effects of 50% and 100% replacement of milk fat for 6% CRW soybean oil oleogel. The fat replacement (50% and 100%) reduced the ice cream’s melting rate, negatively impacting the ice cream overrun. However, the 50% replacement of the traditional lipid phase by CRW oleogel was performed without causing sensory impairment. Thus, this study demonstrates that making ice cream using an oleogel as a partial cream substitute is possible without significant differences in sensory properties [84].

BEW oleogel was also tested as a milk fat substitute in ice cream. The oleogel, in this case, was formed by 7% BEW in camelia oil, and an ice cream control with only camelia oil was also produced. Results showed that among the ice creams, the overrun rate, melting rate, and hardness of oleogel ice cream were mild, and the first dropping time was not significantly different from that of the control, which was much longer than that of camellia oil ice cream. Regarding sensory analysis, the control ice cream had the highest score and was the most accepted by the public. On the contrary, the sensory analysis score of the camelia oleogel cream was less acceptable [85].

A non-wax system using a phytosterol:γ-oryzanol oleogel to replace milk fat in ice cream was studied, presenting high-quality characteristics comparable to those of the milk cream samples, with better overrun and melting starting time [86].

For ice cream, in a summary of the recent findings, waxes such as RBW, BEW, and CRW seem to be able to form oleogels and fully replace the milk fat from a technological point of view. Nevertheless, sensory acceptance was only achieved by 50% replacement.

#### 4.2.2. Cream Cheese

A few studies are available concerning cream cheese’s rheological properties, particularly the critical behavior during heating and cooling when cream cheese is employed in baked goods like cheesecake. However, cream cheese’s rheological and textural characteristics depend on the quantity of saturated and unsaturated fatty acids. Many previous studies reported that cream cheese made from full-fat milk has the most excellent value for firmness, and is less sticky than cream cheeses from low-fat or fat-free milk [39,87,91]. In a study on the application of oleogel in cream cheese production, RBW and EC oleogelators were combined with vegetable oils and other non-fat components. Two RBW and two EC oleogel cream cheese (OCC) products were made and compared to full-fat and fat-free commercial cream cheeses. A cream cheese produced with 100% oleogel had a 25% lower total fat content and a higher UFA content than the full-fat commercial and control samples. In single penetration and strain sweep tests, RBW and EC OCC samples were similar to the control sample in hardness, spreadability, and stickiness. In addition, the OCC samples and the control have similar microstructures, which accounts for their identical textural qualities. These findings characterize the usage of RBW and EC oleogels to replace saturated fat to produce healthy cream cheese formulations [39].

The possible adverse effects of thermal processing during oleogelation and oleogel-based food production were observed by Park et al. [87]. In this experiment, oleogel was made with HOSO and 10% RBW and applied to produce oleogel cream cheese. Then, the laboratory samples’ (HOSO, oleogel, and the oleogel cream cheese) oxidative stability and tocopherol content were compared to the ungelled control and market-available samples. The oxidation study showed that the oleogel sample had more volatile compounds than the HOSO samples, but a negligible difference was observed between the volatile content of oleogel cream cheese and ungelled cream cheese samples. Furthermore, although analysis indicated that the HOSO sample initially had a higher total tocopherol content, oleogel cream cheese showed a comparable total to ungelled control and commercial samples after 14 days of storage. These findings revealed that oleogel maintains its stability during thermal processing and storage, potentially making it valuable for dairy products.

#### 4.2.3. Other Types of Cheese

An additional study by Huang et al. [88] explored the promising use of vegetable oil oleogels instead of solid milk fat in processed cheese products (PCPs). This experiment prepared oleogels using soybean oil as a continuous phase, and two different concentrations of RBW or SFW as oleogelators. The oleogels were then applied to produce oleogel processed cheese products (OPCPs), where the final concentrations of wax were 0.5% or 1%. After that, compositional, rheological, and thermomechanical analyses were done, and the values were compared to the ungelled processed cheese products (UPCPs) and commercial cheese (CC). The results show that OPCPs and the CC samples had equal fat and moisture content, identical hardness, and storage modulus.

Nevertheless, the OPCPs showed a lower level of saturated fatty acids than the UPCPs and CC. Moreover, according to the microscope inspection, OPCPs had a higher oil binding capacity (OBC) than UPCPs. This study reveals the opportunity for oleogels to replace milk fat in high-quality processed cheese products.

Oleogels structuring canola oil with CRW were used as replacements for palm oil in producing vegetable cheese low in saturated fatty acids. Three samples of oleogel were made for this experiment, each using 3%, 6%, and 9% CRW. Equal amounts of palm oil and oleogels were used to produce four samples of cheese (51.0 g). The oleogel imitation cheese samples showed improved viscoelasticity properties. The cheese meltability was unaffected by substituting 3% and 6% oleogels. Additionally, the proportion of saturated to unsaturated fat was considerably reduced from 0.84 to 0.06, demonstrating their nutritional superiority [89].

In the production of pickled or unpickled soft cheese, oleogels were produced using BEW (7%) and olive oil or sunflower oil as a fat replacer [90]. Shortening, oleogels, and milk fat were used to make the cheese samples. After that, the texture and sensory qualities of the cheese samples were evaluated. When oleogels were used, the texture became harder, gummier, and chewier, but the cohesiveness values generally decreased. Cheese made with oleogels showed significantly higher values for all texture parameters, including toughness and chewiness. Although consumers may have a modestly favorable view of the created oleogels for color and taste, excluding texture, the total acceptance ratings suggest that this perception may be minimal.

Most of the studies have used vegetable waxes as structuring agents for cheese and processed cheese. All RBW, BEW, CRW, and SFW waxes and the EC seem to produce similar technological cheeses. Additionally, RBW produced good cream cheese with similar shelf life, and CRW produced soft cheeses with good sensory properties.

### 4.3. Meat Products

Generally, food from animal sources contains a high level of SFC, the main concern for non-communicable diseases. Plant-based oil could be an alternative in processing meat products, but as discussed, some desirable textures and flavors come from SFA. These characteristics are irreplaceable by using pure vegetable oils. However, many studies have reported the effectiveness of oleogels as a saturated fat replacer. When oleogels are applied, they improve the textural and organoleptic properties of the meat products [92,93,94], which have been briefly presented in Table 3.

#### 4.3.1. Meat Patties

In the past few years, research on meat patties and the application of oleogels as a potential animal fat replacer have been conducted. Using HPMC, canola oil was successfully transformed into a solid, like oleogels, and their potential as a replacement for beef tallow in meat patties was evaluated. When beef tallow was replaced with HPMC oleogels, the cooking loss of meat patties was reduced by 50% to 100%. Furthermore, according to the sensory evaluation, 50% replacement of beef tallow had the highest overall acceptability. In addition, the saturated to unsaturated fat ratio was lowered from 0.73 to 0.18 using HPMC oleogels instead of beef tallow, resulting in healthier meat patties [95].

A study also replaced pork patty fat with an oleogel derived from linseed oil (rich in PUFA) [94], in which 25% and 75% of the solid fat in pork patties were replaced with 8% γ-oryzanol:β-Sitosterol oleogel. The fatty acid profile changed when oleogels were added, and the ratio of omega-6 to omega-3 fatty acids dropped dramatically. The results demonstrate that the textural parameters of the oleogel and control patties are identical in hardness, cohesiveness, and chewiness, which is valid for both fat substitution levels. Even though the sensory panel preferred the control samples, the acceptability and preference tests effectively identified products with less oleogel (25%).

#### 4.3.2. Meat Burgers

In a previous experiment, researchers wanted to investigate if they could produce an oleogel out of EC and adipic acid (AA) and employ it in beef burgers [93]. When AA was introduced to oleogels, new intramolecular and intermolecular hydrogen bonds were formed, according to FTIR analysis. Furthermore, differential scanning calorimeter (DSC), X-ray diffraction, and microscopic studies revealed that AA improved the thermal behavior and crystallinity of EC-based oleogels. As a result, the oleogel sample containing EC2%:AA4% presented the maximum oil-binding capacity (97%) and, for this reason, was applied in beef burger recipes as an “optimal” sample. Consequently, the burgers made with EC2%:AA4% oleogel presented an excellent textural profile, color, and flavor.

Impacts of BEW ratio (5, 7.5, and 10%) and cooling temperature (4 and 25 °C) on the characteristics of oleogel structured with sesame oil were evaluated and compared to those of beef flank and shank fats. The 10% BEW oleogel was cooled to 4 °C used to replace 0, 25%, and 50% of the animal fat in beef burgers [96]. The burgers were analyzed for composition, shrinkage, fat absorption, microstructure, texture, oxidative stability, and taste. The oleogels were softer than animal fats, making the raw burgers approximately 50% softer, gummier, and chewier than the control sample. Moreover, the color of the cooked burger barely changed. Therefore, adding oleogel to the beef burger was advantageous since it reduced cooking loss by 11% and fat absorption by 1.6%.

Gómez-Estaca et al. [92] investigated if EC and BEW oleogels with a lipid mix between olive, fish, and linseed oils could be utilized to substitute fat in creating novel fresh meat products. Based on physical-chemical properties (color, thermal qualities, texture, and fatty acid makeup), the oleogels were suitable for their intended usage. When stored at 3 ± 1 °C, they remained stable for at least one month. When the produced oleogels were utilized to replace all of the pork backfat in low-fat pork patties, the burgers became softer and did not change significantly in appearance versus control. Despite significant lipid oxidation, mainly when EC oleogel was utilized, the reformulated burger’s fatty acid profile was much better than the control, with a 3.6-fold increase in the PUFA/SFA ratio and a 23-fold decrease in the n-6/n-3 ratio. In addition, the burgers created with BEW oleogel performed better in a taste test than those manufactured with EC, which performed lower than the neutral point. However, the findings reveal that the BEW oleogel might be utilized to manufacture healthier fresh, low-fat pork burgers with a better fatty acid composition.

#### 4.3.3. Emulsion-Type Sausage

In a study carried out by Barbut et al. [40], different oleogels were made using canola oil, and different proportions of EC and sorbitan monostearate (SMS) and were used to substitute solid beef fat (BF) in frankfurters. The oleogel samples were OG-I (8% EC and 1.5% SMS), OG-II (8% EC and 3.0% SMS), and OG-III (10% EC and 1.5% SMS). Then, 0%, 20%, 40%, 60%, and 80% of the BF was replaced. Compared to the BF control, the high hardness values (texture profile analysis and sensory) of frankfurters manufactured with canola oil alone might be reduced if oleogels were employed instead of BF at all levels. Hardness and springiness were unaffected by the use of OG-I and OG-II. It was often the same as the BF control and less than the canola oil control. As the amount of oleogel replacement increased, losses in the smokehouse yield decreased. Sensory research revealed that while employing canola oil alone increased hardness, structuring the oil (through oleogelation) reduced it to the BF control’s value in all OG-I and OG-II formulations. The meat was significantly less moist when using canola oil and juicier when using more oleogels.

Sunflower oil oleogels and emulsions were used to partially substitute pork backfat in frankfurters by Panagiotopoulou et al. [97]. A total fat percentage of 20% was used in nine frankfurter treatments. The control added 20% pork backfat, while the other eight treatments had 10% pork backfat and 10% oleogel-in-water emulsions (produced with different ratios of γ-oryzanol and phytosterols). The frankfurters did not present variations in pH or oxidation levels. Texture profile research revealed that oleogel-coated frankfurters were no different in any textural parameter from the control. However, emulsion-coated frankfurters exhibited reduced texture parameters such as gumminess, chewiness, and hardness values. The sensory results revealed that all treatments presented similar overall acceptability to the control except for some emulsion treatments. Pork backfat can be partially substituted with oleogels without altering the frankfurter’s overall physicochemical and sensory qualities.

An experiment by Wolfer et al. [98] investigated the application of RBW oleogel on the quality of pork frankfurters. This research examined how the organoleptic qualities of finely ground frankfurter-style sausages changed when pork backfat was replaced with RBW oleogels. The lipid substitutes used in the Frankfurter recipe were the control with pork fat (PF), soybean oil, 2.5% RBW oleogel, and 10% RBW oleogel. TPA analysis showed that oleogel treatments were comparable to pork fat treatments in stiffness, chewiness, and springiness. In addition, a sensory test revealed that while replacing PF did not change the smell of cured frankfurters, it significantly reduced their flavor. In addition, lipid oxidation significantly differed in samples containing PF and 10% RBW oleogel, and RBW oleogel showed greater TBA values.

By monitoring and measuring their impacts on product processing and quality features, Tarté et al. [99] set out to investigate the utilization of RBW/soybean oil oleogels prepared using high-oleic or standard soybean oil to substitute animal/saturated fat in bologna-type sausage. In order to substitute 41.9% of the control (PF) product’s total fat, oleogels prepared with 90.0% or 97.5% of either oil, with RBW as the oleogelator, were added to bologna-type sausage manufactured with mechanically separated chicken. Although there were no changes in yield, raw batters for bologna using either PF or oleogels were more stable than those with soybean oil. Aroma, taste, texture, and moistness did not vary in sensory ways, although the PF-containing product’s color was more intense. Both high-oleic and regular soybean oil were successfully used to make oleogels, which produced bologna products with comparable quality and organoleptic characteristics, proving that they may be used interchangeably in this situation. However, using high-oleic soybean oil will give the sausage a better fatty acid composition. Once soybean oil is used instead of native liquid soybean oil in a lower-protein sausage like the one created in this research, the outcome may be firmer batter and a softer product texture.

#### 4.3.4. Fermented Sausages

The impact of partial replacement of PF by olive oil oleogel on the technical and dietary qualities of fermented sausages was investigated. Moreover, the authors simultaneously tested the substitution of sodium chloride (NaCl) for potassium chloride (KCl) [100]. Four different fermented sausages were tested: control (C) contained pork backfat (18 g) and sodium chloride (1.5 g per 100 g), treatment (N), which substituted 50% of the NaCl with KCl, treatment (O) contained 50% oleogel instead of PF, and treatment (D), which had 50% KCl and oleogel simultaneously. Treatment (D) showed less weight loss and more water activity. A nine-person trained panel used sensory assessment to identify changes between treatments regarding color and textural features, but not taste, bitterness, or salt. Treatment (O) was rated as the second-best treatment by a 54-consumer panel regarding personal choice (C). The findings demonstrate that fermented sausages with a better nutritional profile may be produced that are both microbiologically and sensory-safe.

In an earlier study, Pintado and Cofrades [101] assessed the acceptability of BEW oleogels and emulsion gels made with a lipid combination of olive and chia oils as PF substitutes for producing dry fermented sausages. An oleogel and an emulsion gel (EG) were produced using solid-structuring oil systems. Ninety percent of oleogel composition was oil, compared to 45% in EG. The lipid phase was made of 80% olive oil and 20% chia oil. This study showed that oleogel and EG sausages presented a better fatty acid profile and a 12-fold lower PUFA n-6/n-3 ratio than the control. In addition, reformulated samples had a satisfactory oxidative condition during refrigerated storage, regardless of the structural technique utilized as an animal fat substitute. The technical features and microbiological status of oleogel and EG were essentially the same; therefore, choosing one over the other would be determined by considerations other than how the product behaves.

### 4.4. Breakfast Spreads

Many studies on spreads and margarine have been done using vegetable waxes as oleogelators, which are summarized in Table 4. Initially, Hwang et al. [102] evaluated SFW, RBW, and CLW (2–6%) soybean oil oleogel suitability for incorporation into spreads and margarine (20% water). Using CLW in the formulation, phase separation in the emulsion was observed. On the other hand, RBW oleogel used in margarine lowered the firmness. SFW oleogel in the margarine formulation presented similar firmness as the margarine with 18–30% hydrogenated soybean oil. Firmness similar to the commercial spread was achieved with 2% SFW, and firmness similar to commercial margarine was achieved with about 10% SFW.

Further evaluation of SFW oleogel with 12 different vegetable oils was performed in a margarine study [103]. Margarine with oleogels containing 3% SFW was firmer than conventional spreads, whereas margarine made from 7% SFW was softer. Margarine firmness did not correlate with the oil fatty acid composition, implying an advantage for developing margarine and spread products with nutritional properties, since the higher polyunsaturated fatty acid content would not necessarily reduce the firmness of margarine within this range of UFA.

Additionally, this group recently evaluated the possibility of producing wax oleogel margarine using hempseed oil [104]. They used SFW and RBW, with contents of 3, 4, or 7%. Using wax oleogels, the required firmness can be achieved in commercial spreads with less than 3% wax, while for the stick margarines, the required firmness cannot be reached even with 7% wax [104]. BEW oleogel (4–10%) was also evaluated as an alternative to producing wax oleogel-based margarine for a different group [105]. The results showed that the oleogel margarine hardness increased as the BEW ratio increased, due to the denser droplets and crystal network structure formed. In addition, all samples formed the β’ polymorphic form. However, the oleogel-based margarine samples presented a melting temperature of over 40 °C, representing a poor mouth-melting characteristic [105].

The sensory profile of wax spreads with BEW and SFW oleogels structured in hazelnut and virgin olive oil was assessed [106]. According to the findings, both forms of oleogels are appropriate as alternative goods (structurally and thermally). Using thirteen sensory description terms (spreadability, hardness, liquefaction, milky, grassy, fatty, rancid, sweet, salty, waxy, mouth coating, grittiness, and cooling), a sensory panel described these oleogels. Consumer tests using hedonic scales (appearance, odor, flavor, and spreadability) revealed that these oleogels show potential as ingredients of spread and/or butter substitutes. One of the most impressive findings by the consumer survey was that over half of the consumers would buy or try the oleogel products once before buying them.

Another type of wax, shellac wax (SLW), was also evaluated in spreads. Using 2 or 4% shellac wax and 20% water showed a more fluid consistency, while the emulsion containing 6% shellac wax produced a solid-like behavior, with the loss and storage modulus (G’ and G”) reaching 60,000 Pa [29]. Recently, a two-factorial design was conducted to find the optimum formulation for a spread formulation with SLW emulsion. The analysis of the variance of two factors (wax and water concentration) confirmed that the texture was affected by wax concentration, the rheology and stability by both the considered numeric factors and their interaction, and the color by both factors. The emulsions containing 7% SLW oleogel performed like the strongest systems, with high a viscoelasticity and cross-over point. All oleogels and emulsions showed an oil binding capacity higher than 99.88%. Therefore, a formulation containing 4.29% SLW and 24.13% water was appropriate as a low-fat spread. However, the formulation possibilities are vast, since the product’s functionality is fulfilled even by emulsions containing 60% water and 7% SLW, 40% water and 7% SLW, or 60% water and 5% SFW [107].

In order to reduce the amount of wax or find some specific physical properties, combinations of structuring agents have also been evaluated. CLWX (2.66%): MAG (1.77%) HOSO oleogels were used as margarine’s lipid phase. The color results were similar for the oleogel margarine compared to commercial margarine. Oleogel margarine presented a structure more resistant to temperature oscillations and an overall softer product. However, the firmness and spreadability of the oleogel margarine were lower, and consumers easily detected the sensorial difference between samples, mainly concerning the parameters of taste, texture, and overall impression [108].

An oleogel containing 10% BEW combined with 3.15% sodium caseinate, 0.5% guar gum, and 0.22% xanthan gum (XG) was developed as a substitute for palm oil and partially hydrogenated palm olein oil in margarine with 70% fat. The replacement of 100% of the partially hydrogenated palm olein oil (PHPO) and 25% of the palm oil (PO) with the oleogel, and the replacement of 100% of the PHPO with hydrocolloid-based oleogel resulted in similar texture and rheological properties in margarine compared to the commercial control margarine. In addition, the oleogel and hydrocolloid-based oleogel samples presented a higher melting point and a lower content of SFC than the commercial control sample [109].

BEW was also blended with China lacquer wax (ZLW) (5 and 10% total), and margarine was prepared with an oleogel containing 5% structuring agent (BW50%:ZLW50%) and fast cooled, presenting similar melting and texture properties at 37 °C compared to commercial margarine. The interaction of the BEW and ZLW crystal networks governed the crystallization network of oleogel margarine. These authors have also evaluated the possibility of producing the oleogel margarine with fast cooling. Nevertheless, the oleogel margarine produced with a rapid cooling process had much higher hardness and spreadability than those produced in a low cooling process [105].

For margarine and spreads (water in oil), several studies were able to fully replace high saturated fats in these products. Mostly positive results were found with waxes. From a technological point of view, better physical properties were found for SFW and SLW waxes. Although no sensory analyses have been performed for them, they produced good margarines with a lower wax concentration.

### 4.5. Chocolate and Confectionary

Current application of oleogel in chocolate and confectionary is describe below and has been summarized in Table 5.

#### 4.5.1. Chocolate

Chocolate is a product widely manufactured and consumed, traditionally produced with cocoa butter, whose melting point is 34 °C, promoting melting as soon as it reaches body temperature (37 °C). The challenge is when chocolate is manufactured or sold in warmer climates. Stortz and Marangoni [118] developed chocolate with heat resistance using EC as a structuring agent of the lipid phase, preventing the structure from collapsing during heating. Nevertheless, EC would need to be heated to around 145 °C for gelling, but this temperature would prevent its application in chocolate. Thus, another study proposal was to develop a low-temperature method that would enable the application of the structuring agent [41]. EC was dissolved in ethanol (EtOH) at room temperature, forming a solution applied to the melted chocolate. After cooling and crystallizing the chocolate, it was reheated at controlled temperatures to evaporate the solvent without melting. Other solvents were also tested, such as limonene, which did not sufficiently solubilize the EC, and ethyl acetate (EA), which dissolved the EC and evaporated faster, but showed less heat resistance than chocolate with the EtOH solution. A possible explanation would be that lecithin phospholipids are not soluble in EA, and perhaps removing them from the formulation would result in greater product heat resistance. The EC dispersion limit in EtOH was more relevant than the type of chocolate (white, milk, dark), as with 20% dispersion, all chocolates gained more heat resistance than with 25%. Evaporation of the solvent did not affect the texture of the product. EtOH, even without EC, played a role in developing heat resistance and containing oil loss, probably due to the dissolution of some phospholipids in soy lecithin (ordinarily present in chocolate compositions as an emulsifier). It was concluded that the manufacture of chocolate with heat resistance was possible with 1.9% EC, and that the resistance can be adapted depending on the fraction of the solution applied.

Recently, oleogels were developed using EC and other alternative oleogelators, such as MAG and β-sitosterol/lecithin to partially (50%) and completely (100%) substitute the cocoa butter (CB) in chocolates. Only MAG could replace CB at 100% with a solid-like texture. However, the existence of significant volumes of liquid-form UFA produced a soft texture and low SFC. With the 50% replacement, the EC oleogel containing chocolate had more outstanding toughness and yielding stress than the chocolates made with MAG oleogel or β-sitosterol/lecithin oleogel, which was mainly due to the increased SFC values. However, the thermal and polymorphism characteristics of all oleogel chocolates were similar to those of dark chocolate [110]. Therefore, it will take further research to completely replace the CB in chocolate.

#### 4.5.2. Chocolate Paste and Spreads

Patel et al. [29] studied the behavior of oleogel made with rapeseed oil structured with shellac in chocolate paste (replacing 27% of the palm oil). Even after storing the paste made with oleogel at 30 °C for four weeks, there was no oil loss, which shows shellac’s effectiveness as an oil binder. The sample’s viscosity was comparable to that of the control sample; however, the yield strength was higher for the oleogel paste because of the significant interaction between the dispersed particles (sugar) with lower SFC. Furthermore, the viscosity of the oleogel paste decreased with shear, and partially recovered with the interruption of flow, which shows the partial breakdown of the structure, a characteristic of oleogels made with shellac [29].

To replace 50% of the palm oil, oleogels (5%) of MAG, BEW, and propolis wax (PPW) were utilized in chocolate spreads (CS). Findings showed that in PO oleogel systems, the MAG, BEW, and PPW chemical natures contributed to creating various crystalline networks. All samples did, however, create a β′-polymorphic form. The mechanical parameters increased in the following order: PPW < BEW < MAG in CS made with PO oleogels. This pattern may be explained by the chemical makeup of oleogelators and the physical linkages created in the samples. Due to the oil release being less than 6% after centrifugation, all samples had a high OBC [111].

Recently, oleogels containing two oils, olive and sunflower, as well as HPMC and XG as structural agents, were used to create CS. The results demonstrated that the network created by HPMC and XG gave the sunflower oleogel chocolate spread consistency. They created an impermeable barrier that was both hard and thick, which reduced the mobility of the molecules within the matrix and prevented linkage of the solid particles. Although replacement at 50% produced homogenous spreads that resembled the control spread, replacement at 100% produced less homogeneous products. The chemical compatibility of oleogel and CB may be blamed for this tendency. When chocolate spread was substituted for oleogel at 50%, sensory testing revealed that it had the same “creamy look”, “creamy texture”, and “cocoa flavor” as the control spread. When respondents were asked to assess the 100% replacement, they cited “lumpy look”, “lumpy texture”, and “thick texture”. The outcome was consistent with observing undissolved particles in this sample on a microscopic scale, and the completely solidified oleogel was also consistent with its “gummy texture” and “oily texture” [112].

In a previous experiment, chocolate butter was made using walnut oil, CLW, and MG (10%). Oleogels and cocoa butter spread with an oleogel basis exhibited considerable gel behavior (G’ > G”). In comparison to the MAG oleogel-based spread, and similar to the control sample, the product formed after the usage of CLW was significantly thicker and more spreadable. In addition, each sample displayed a great OBC [113].

Corn oil was used to create the oleogel phase before chocolate spreads were created using water-in-oleogel (W/O) emulsions with the following ratios: 0:100, 45:55, 50:50, and 55:45 (water:oleogel). It was discovered that the reference sample, which contained 100% oleogel, had an emulsion with a water:oleogel ratio of 45:55, which produced chocolate spread with water activity, Casson viscosity, yield stress, a linear viscoelastic region, firmness, and spreadability that was quite similar to the reference sample. In addition, more open spaces and voids were seen between the fat droplets as the water concentration rose, and the interactions between the droplets and the fats were less tightly packed and noticeable. Additionally, the low-fat chocolate spread had sensory evaluations and acceptance levels equivalent to the full-fat samples [114]. This MAG emulsion oleogel was the only study that met the physical and sensory properties to replace palm oil in chocolate paste; nevertheless, the amount of MAG was relatively high.

#### 4.5.3. Filling Creams

At three degrees of replacement (17, 33, and 50%), PO was swapped out for RBO-BEW oleogels in fillings. BEW oleogel’s crystallization ability has been shown to aid in the immediate chilling step’s gelation of the hybrid mixes. Wax-based oleogels could only substitute up to 17% of the original formulation’s PO, leaving the system’s gel strength comparable to the control. With increasing oleogel percentage, the dilution effect and melting enthalpy were reduced. However, the final products had an SFC below 2.0%wt at body temperature, which points to a mouthfeel free of wax [115].

Tanti et al. [9] tested the total or partial replacement of the shortening that makes up cookie fillings with HPMC and MC oleogels. In order to facilitate air incorporation into the cream, the HPMC and MC solutions were first foamed by stirring, then frozen, lyophilized, crushed, and then mixed into the oil. The greater the replacement of shortening by oleogel, the greater the nutritional quality of the product, and in the long term, the greater the oil binding capacity, which results in a more stable product than the commercial one. As for texture, the partial replacements of 50% and 75% generated products which were very similar to the commercial ones. In these proportions, the hardness and viscoelasticity values are statistically equal, as is the adhesiveness in 75% of both oleogels, the gumminess in 50% of the HPMC oleogel, and 75% of the MC oleogel. This demonstrates the ability of these oleogels to replace both the lipid phase and thickening additives sometimes used in the industry, such as gums, starches, and gelatin [9].

MAG (10%) oleogel was also tested in fillings for cookies. Besides the full replacement, authors also changed the total amount of lipid phase in the filling (22–40%). The oleogel concentration greatly influenced the technical characteristics of the filling. When the amount of oleogel content rose, the ability of fillings to bind oil declined. Reduced hardness resulted from adding more oleogel to the formulation, but increased adhesiveness and cohesiveness were created instead. Compared to the viscoelastic moduli values determined in a commercial filling cream used as a reference, oleogel fillings made with 26% oleogel did not vary noticeably. A decreased oil loss was also maintained after 21 days of storage when they were combined into cookies [116].

The viability of using oleogels made from binary mixes of CLW and MAG in various ratios with canola oil was also investigated. The CLW:MAG 6:4 oleogel created filling creams with comparable textural characteristics as shortening, and these textural characteristics might lessen the oil separation issue from the samples when utilizing this CLW:MAG proportion. In addition, the MAG oleogel showed a superior aerated structure and tomographic analysis, indicating the importance of MAG for these technological properties in fillings. The CLW oleogel showed intermediate results, except for the aeration capacity. Correlation analysis showed that the morphological properties of the filling are correlated with specific gravity, and this property is strongly affected by the level of SFA in the formulation [30].

As a substitute for shortening in fillings, a more complicated system comprising wax (CLW), MAG, and high-melting TAGs (HF) in SBO and HOSO was also investigated [103]. In this instance, the oleogel was always a ternary-structured system with varying amounts of CLW:MAG:HF (5–10% total). The percentage of MG and HF had a substantial effect on the product’s structure, as the higher the concentration (3%) of oleogelators, the better the creation of the structured network, with excellent aeration, higher firmness, and reduced oil loss in comparison to the shortening filling. Samples at ratios of 3:3:3 and 1:4:5 (CLW:MAG:HF) exhibited comparable physical attributes to the control sample. Similar oil loss at T0 (~4.5%) and microstructure before and after temperature oscillations were seen in these samples, but with somewhat reduced consistency and adhesiveness. The authors hypothesized that the MAG and HF strengthened the CLW’s greater structuring power by building a network resistant to stirring and heat changes, making this system a viable substitute for shortening in cookie filling applications [117].

In fillings, a common finding between several studies was that some fatty acids oleogelators are needed to aerate the product. With this in mind MAG, TAGs, or their combination with others oleogelators, as described for CLW:MAG, were good candidates to fully replace shortening in this type of product. Nevertheless, no sensory study has been done for this product so far.

### 4.6. Peanut Butter

Whole peanut butter is a product known for presenting oil separation, and to control this natural process, stabilizers are added to the product. Preventing the separation of unbound oil reduces viscosity and improves texture and spreadability. These stabilizers are often entirely or partly hydrogenated PNO, SBO, cottonseed, or RSO, ranging from 0.5% to 5.5% of peanut butter’s total weight [119].

Some oleogel applications in peanut butter are presented below and summarized in Table 6. The applicability of HPMC and MC as peanut butter (PB) stabilizers was investigated. As these structural agents are hydrophilic, the authors have investigated methods for maximizing their utilization. Without this templating method, HPMC and MC did not influence the consistency and stability of PB. An alternate spray drying method was explored; however, it was discovered that high inclusion levels were necessary for any stabilization effect, which is undesired. This approach decreased oil loss in PB stabilized with freeze-dried HPMC and MC (0.2%), while no oil loss was seen at 2.2%. With the addition of >1% HPMC/MC, PB had a shelf life of at least six months. The peanut butter’s textural quality was evaluated using a penetration test. Including freeze-dried HPMC/MC enhanced sample hardness and adhesiveness, emulating the qualities of conventional PB stabilized with hydrogenated plant oils [120].

Winkler-Moser et al. [119] evaluated natural waxes, including BEW, CLW, RBW, and SFW (0.5–2%) as active stabilizers in peanut butter. Winkler-Moser and colleagues discovered that natural waxes might have a significant commercial value as a replacement for hydrogenated vegetable oil (HVO). SFW exhibited the greatest OBC, the lowest oil separation percentage after six months of storage, and the highest hardness among the natural waxes. In addition, at over 1.0%, all four waxes inhibited long-term oil separation of PB in a manner comparable to a commercial stabilizer [119]. This group examined the sensory impression of the texture, spreadability, mouthfeel, and taste characteristics of PB samples modified by the various waxes at different levels (BEW, CLW, RBW, and SFW, 1–2%). The waxes and their proportions in the mixture considerably affected the look, spreadability, hardness, mouthfeel, and taste characteristics. The samples with 1.5–2.0% CLW or 1.0–1.5% RBW showed the fewest visual and textural variations from the reference samples.

Nonetheless, an off-flavor was linked to CLW levels of 1.5% or above. RBW showed the least taste variation compared to the standard sample. Generally, samples stabilized with 1% to 1.5% RBW had the highest scores across reference samples. The response to CLW, RBW, and SFW (which were solely examined for appearance and spreadability) show that the proportions of these waxes might be adjusted in various products to obtain a product with the desired texture, taste, and oil loss stability. These samples were evaluated in an unsweetened product; hence, sweetness/bitterness and any off-flavors may be reduced in commercial spreads or other foods that add sugar or different flavors/sweeteners [121]. Ferdaus et al. [122] tested RBW, BEW, and CRW as oil stabilizers in peanut butter. In this study, more physical properties of peanut butter were evaluated. RBW and CRW showed better viscoelastic and oil binding capacity, regardless of wax concentration (1, 1.5, or 2%).

Further storage evaluation then proceeded with 1% RBW and CRW. Regardless of the mixing method, RBW was the superior stabilizer, comparable to commercial peanut butter regarding spreadability, hardness, and OBC (natural and creamy) [122]. Hence, the findings show that RBW oleogel is a feasible substitute for HVO as a stabilizer.

Cellulose derivatives and waxes are good replacements for the current fully hydrogenated stabilizers used in peanut butter, particularly HPMC, MC, and RBX. RBW, in particular, is already sensory and technologically tested.

### 4.7. Frying Medium

Oleogels have a potential application in the deep-frying section regarding less oil absorption, as shown in Table 7. Lim [123] analyzed the alternate use of oleogel in deep-fried quick noodles. Soybean oil was combined with CRW to form a solid-like oleogel for quick-fried noodles as a substitute for a high-SFA deep-frying medium. The viscosity of the oleogel changed more rapidly with the temperature as amounts of CRW increased. As a result, the noodle samples, cooked in oleogel frying medium, absorbed about 16% less oil than the ones fried in PO and SBO. On the contrary, the texture of the noodles remained unchanged. Compared to PO-fried noodles (54 g/100 g), oleogel-fried noodles had much less SFA (19 g/100 g).

Oleogel was used as a frying medium to produce low-fat, fried Indian snacks called mathri [124]. CRW and SBO were blended in three quantities (5%, 10%, and 15%) to produce oleogel, which was subsequently utilized as a frying medium and evaluated. Mathri cooked in oleogel maintained more moisture, had superior sensory attributes, and a lower oil absorption rate than mathri fried in a traditional way (SBO). Oleogel used samples of mathri with a lower breaking strength and higher moisture content than those cooked in SBO. Oleogel-fried mathri absorbed 27.7%, 22%, and 19.3% less oil than the traditional fried product. To address the demand for low-fat meals among health-conscious customers, oleogel may be used to manufacture mathri with less fat and fewer calories.

In contrast to the sunflower oil (control), BEW oleogels were made with 3% and 8% [125]. Oleogels were completely solid at ambient temperature and completely liquid when heated for frying. The results revealed that oleogel-fried strips absorbed substantially less oil (11.97% and 12.07%) than the control sample (15.20%). The samples cooked in oleogel were firmer, springier, and gummier than the control sample, according to the fried potato sample’s textural character. Oleogel-fried potatoes had better sensory ratings, according to sensory analysis. The most excellent overall acceptability score (8.50) was achieved by the potatoes that had been cooked in the 8% BEW oleogel sample. Overall, this research showed that frying potatoes in oleogels resulted in decreased oil absorption and better texture and sensory evaluations.

As a frying medium, although wax oleogels were the only ones texted, they showed solid and positive attributes, specially the CRW and BEW, offering an excellent technological and sensory alternative to palm oil.

## 5. Conclusions

As shown in this review, oleogels have been applied in different types of food, such as confectionary, dairy, meat, bakery, etc. Several oleogelator and oil combinations have been tested according to their textural properties and sensory attributes on different food products, which showed oleogel’s potential as a solid fat replacement. The used oleogelators and their concentrations played a major role in the final application due to their interactions with other ingredients, such as water, sugar, etc. Most of the applications studied MAG and vegetable waxes as oleogelators. They have been viable alternatives, especially in baked products and margarines/spread, where some successful full-replacements, physical, chemical, and sensory properties were found. In meat products, the studied oleogels are also advanced, and polymers and proteins have shown some auspicious results. Although for the confectionary and dairy products there are some promising results been published, such as EC and MAG in chocolates, waxes in ice cream and chocolate paste, and MAG in fillings, more studies confirming these findings regarding shelf life and sensory acceptance are needed if the goal is a 100% replacement. Oleogels have also been shown, as observed in this review, as excellent alternatives to stabilizers in peanut butter, where RBW and cellulose derivatives have been able to replace the hydrogenated stabilizers previously used, and still meet sensory and shelf life requirements. Moreover, as a frying medium, BEW and CRW were shown as great oleogelators to form oleogels to replace palm oil as a frying medium for several products. Though many studies have already proven the positive applications of oleogels in food products, in order to start the commercial and industrial use, more studies on scaling up this technology must be performed.

## Figures and Tables

**Table 1:** Application of oleogels in baked products.

Baked Foods	Oleogelator	Liquid Phase	Major Outcomes	Reference
Cookies	CLW * (3 or 6%)	CNO **	Reduced G’ and G” (viscoelastic parameters) of the cookie doughs. Desirable spreadable property. Cookies with soft eating characteristics.	[66]
	CLW (3 or 6%)	CNO	Cookie dough shows higher extensibility and lower hardness. Sensory acceptable dough and cookie properties. Up to 70% replacement.	[67]
	CLW or CRW (2.5 or 5%)	SFO	Oleogels produced softer cookies than saturated fat oils (SFO). Cookies with a more complex texture and more spreadable than the samples with SFO. Oleogels enhanced cookie quality.	[68]
	CLW, CRW, BEW, RBW (1–10%)	RBO	BEW and RBW oleogel cookies presented lower hardness and L*. Crispness values were similar to the commercial. Cookies with 70% RBW (6%) oleogel received highest sensory score.	[25]
	RBW, BEW, CLW and SFW (2, 4, 6, 8, 10%)	OO, SBO, FXO	SFW and RBW oleogels presented the greatest hardness to the cookie dough. Cookies prepared with 100% oleogels showed similar properties to cookies made with margarine.	[69]
	RBW, BEW, CLW, and CRW (3, 5, 7 and 9%)	CO	RBW and CLW produced soft cookies with porous structures. Oleogels with lower SFC (4.5–11%) and higher β’ crystals, producing cookies with a similar hardness to shortening.	[70]
	HPMC, MAG, RBW, BEW, and SSL (6%)	CO	MAG and RBW oleogels produced similar hardness, porous structure, and L*, a*, b* like shortening-based cookies. MAG and RBW oleogel-based cookies exhibited identical rheological and sensory properties like shortening. The higher the content of β′ crystal and SFC in oleogels, the lower the cookies’ hardness.	[71]
	MAG, SSL, PGE, SPAN 60 (3–18%)	CO	MAG and SPAN oleogel-based cookies show similar hardness and color to those of shortening. Emulsifier-based oleogels resulted in softer cookies.	[72]
	EC, MAG, SFW (2.5–10%)	RSO	Reduction of saturated fatty acids. Oleogel-based cookies were acceptable to consumers.	[73]
	BS and MAG (5 or 10%)	Crude or refined SBO	Cookies made with oleogels showed overall equivalent quality to those prepared with vegetable shortening.	[74]
	MAG and HPMC (0–10%)	SBO	MAG concentration improved the hardness, fracturability, and chewiness, while cookies’ cohesiveness, springiness, and gumminess decreased. 50% replacement was viable.	[75]
Cake	HPMC (4%)	SFO	Reduce steady shear viscosity and viscoelastic parameters of the muffin batters. Increased oleogel percentages showed larger pore sizes in batters and muffin cakes.	[76]
	MAG (4, 7, and 10%)	HOSO	Greater spreadability and a higher specific volume in oleogel-based muffin cakes. Oleogel cake showed a 50% reduction in oil migration after ten days of storage. Oleogel positively impacted the nutritional profile of muffin cake.	[26]
	MAG (6.6%)	HOSO	The sensory analysis showed that 100% oleogel-based muffin had better color, texture, and overall acceptability.	[77]
	MAG and HPMC (0–10%)	SBO	Oleogel significantly improved the hardness and chewiness of cakes. 50% replacement of the shortening was possible.	[76]
Sponge cake	CLW (5%)	CO	Increased oleogel resulted in lower hardness and improved cohesiveness. Increased digestibility of cake starch.	[78]
	SFW, BEW, and RBW (1, 5 and 10%)	CNO	Oleogels stabilized air bubbles in the cake. Oleogel cake samples showed lower oil leaping than those made with pure CNO. The sensory tests scored the quality of the oleogel-based sponge cakes similar to the CNO.	[43]
Plum cake	BEW (3%)	OO	Decreased hardness in plum cake with increased oleogel and whey proteins. Aeration of the batter enhanced and increased the porosity of the cake. Oleogel-based cake samples showed the highest moistness. The sample containing 76.98% oleogel, 7.28% emulsifier, and 15.73% whey proteins produced the best cake.	[10]
Bread and cracker	MAG or RBW (10%)	HOSO	MAG oleogel showed identical qualities to the sample with shortenings in bread. Bread sample with MAG oleogel exhibited a softer crumb firmness. MAG oleogel-based crackers showed reduced hardness.	[79]
Biscuits	CRW, BS, BEW, LE, MAG (10 or 16%)	SFO	Biscuit samples with BS:BEW mixture showed the lowest oil loss (<3.2%). The slightest color difference was found in the BS:LE sample. Oleogel biscuit presented similar storage modulus G’ and loss modulus G” were similar.	[80]

* CLW: candelilla wax; CRW: carnauba wax; BEW: bees wax; RBW: rice bra wax; SFW: sunflower wax; HPMC: hydroxypropyl methylcellulose; MAG: monoglycerides; SSL: sodium stearyl lactate; SPAN60: sorbitol monostearate; EC: ethylcellulose; BS: β-sitosterol; LE: lecithin. ** CNO: canola oil; SFO: sunflower oil; RBO: rice bran oil; OO: olive oil; SBO: soybean oil; FXO: flaxseed oil; CO: corn oil; RSO: rapeseed oil; HOSO: high oleic sunflower oil.

**Table 2:** Application of oleogels in dairy products.

Dairy Foods	Oleogelator	Liquid Phase	Major Outcomes	Reference
Ice cream	RBW (10%) *	HOSO **	Ice cream air bubbles were smaller in the oleogels formulations than in the control sample. Similar fat destabilization to the control. Lower melting properties of the oleogel ice cream.	[11]
	RBW, CLW, and CRW (10%)	HOSO	RBW shows higher meltdown stability and fat aggregation. Oleogel containing 15% RBW in ice cream showed better form stability.	[83]
	CRW (6%)	SBO	Oleogel reduced the melting rate of ice cream. The ice cream with 50% CRW oleogel showed the same acceptance level as the control.	[84]
	BEW (7%)	CMO	The first dropping time was not significantly different from the control ice creams. Sensory analysis showed that the control samples had the highest score and were the most accepted by the public.	[85]
	PHY and γ-orizanol (8–12%)	SFO	12% oleogel showed better quality characteristics ice cream. Improved overrun and melting properties.	[86]
Cream cheese	RBW and EC (10%)	HOSO and SBO	Cream cheese samples containing oleogel had a 25% lower total fat than full-fat commercial products. Oleogel cream cheese was similar to the full-fat commercial control sample in hardness, spreadability, and stickiness. Similarities attributed to similar microstructure found.	[39]
	RBW (10%)	HOSO	Oleogel maintains its stability during thermal processing and storage. Cream cheese network enhanced the oxidative stability of oleogel cream cheese.	[87]
Processed cheese products	RBW (0.5 or 1%)	SBO	Improved oil binding capacity. Oleogel cheese and the commercial samples had equal fat and moisture content, identical hardness, and storage modulus.	[88]
Imitation cheese	CRW (3, 6 and 9%)	CNO	Oleogel imitation cheese samples showed improved viscoelasticity properties. The cheese meltability was unaffected by substituting 3 or 6% oleogels.	[89]
Pickled or unpickled soft cheese	BEW (7%)	OO or SFO	In oleogel-added sample, the texture became harder, gummier, and chewier. The overall acceptability of SFO oleogel sample was close to the standard sample.	[90]

* CLW: candelilla wax; CRW: carnauba wax; BEW: bees wax; RBW: rice bran wax; EC: ethylcellulose; PHY: phytosterol. ** CNO: canola oil; SFO: sunflower oil; OO: olive oil; SBO: soybean oil; HOSO: high oleic sunflower oil; CMO: camelia oil.

**Table 3:** Application of oleogels in meat products.

Meat Products	Oleogelators	Liquid Phase	Major Outcomes	Reference
Pork patties	BS *: γ-oryzanol (8%)	LSO **	Increased fatty acid profile without changing the physical and sensory attributes. The patties made with oleogels presented a similar hardness, cohesiveness, and chewiness to the control in both fat substitution levels. The acceptability and preference tests effectively identify products with less oleogel (25%).	[94]
Beef Patties	HPMC (2, 4, and 6%)	CNO	Cooking loss has been reduced by 50% to 100% in the patties. The highest overall acceptability was obtained in the sample with 50% replacement of beef tallow.	[95]
Meat burger	EC and AA (0–6%)	SBO	No adverse effect on texture and organoleptic characteristics. The burgers made with EC2%/AA4% oleogel had an excellent textural profile, color, and flavor.	[93]
Beef Burger	BEW (5, 7.5, and 10%)	SSO	Oleogel burgers were approximately 50% softer, gummier, and chewier than the control sample. The oleogel burgers show a reduction of 11% in cooking loss and fat absorption by 1.6%.	[96]
Pork burgers	EC and BEW (11%)	OO, LSO, FO mixture	Burgers became softer and did not alter significantly in appearance compared to the control. BEW oleogel performed better in a taste test than those manufactured with EC.	[92]
Beef frankfurters	EC (8 or 10%) and SMS (1.5 and 3%)	CNO	Hardness and springiness were unaffected using oleogel. As the amount of oleogel replacement increased, the losses in the smokehouse yield decreased. Samples were juicier when using more oleogels.	[40]
Pork Frankfurter sausages	PHY:γ-oryzanol (10 or 20%)	SFO	Frankfurters did not show differences in pH or oxidation levels. Oleogel-coated frankfurters were no different in any textural parameter from the control.	[97]
Pork frankfurter	RBW (2.5 or 10%)	SBO	Oleogel treatments were comparable to pork fat treatments in terms of stiffness, chewiness, and springiness. Replacing pork fat did not change the smell of cured frankfurters, it did significantly reduce their flavor. RBW oleogel showed greater TBA values in frankfurter samples.	[98]
Bologna-type sausage	RBW (2.5 or 10%)	SBO or HOSBO	Aroma, taste, texture, and moistness did not vary in sensory ways. HOSO provided a sausage with a better fatty acid composition. Oleogels produced stable batters and a softer product texture.	[99]
Fermented sausages	MAG (15%)	OO	Oleogels produced sausages that were both microbiologically and sensory-safe.	[100]
Dry fermented sausages	BEW (10%)	OO and CHO	Oleogel sausages presented a better fatty acid profile and a 12-fold lower PUFA n-6/n-3 ratio than the control. Reformulated samples had a satisfactory oxidative condition during refrigerated storage.	[101]

* BS: β-sitosterol; HPMC: hydroxypropyl methylcellulose; BEW: bees wax; RBW: rice bran wax; EC: ethylcellulose; PHY: phytosterol; MAG: monoglycerides; AA: adipic acid; SMS: sorbitan monostearata. ** LSO: linseed oil; CNO: canola oil; SFO: sunflower oil; OO: olive oil; SBO: soybean oil; HOSO: high oleic sunflower oil; HOSBO: high oleic soybean oil; SSO: sesame oil; CHO: chia oil.

**Table 4:** Application of oleogels in breakfast spreads.

Spreads	Oleogelators	Liquid Phase	Major Outcomes	Reference
Margarine and spreads	SFW * (2–10%)	SBO **	Commercial spread and margarine firmness were achieved with 2% and 10% SFW, respectively.	[102]
Margarine and spread products	SFW (3, 5, and 7%)	Vegetable oil	Firmness of margarine was higher than commercial spreads and lower than commercial stick margarine.	[103]
Margarine	SFW, RBW, BEW or CLW (3,4 or 7%)	HPO	Commercial margarine firmness was achieved with less than 3% wax, while the firmness of stick margarine was not achieved even with 7% wax.	[104]
Margarine	BEW (4–10%)	LSO	Oleogel-based margarine had a high melting peak at 40 °C. Poor mouth-melting characteristics. Margarine containing oleogel hardness increased as the wax concentration increased.	[105]
Spreads or butter	BEW or SFW (5%)	OO and HZO	Oleogels were structurally and thermally appropriate as alternative spread products. Sensory tests revealed that these oleogel products could be used as alternatives.	[106]
Spreads	SLW (2 or 4%)	RSO	Same characteristics as commercial spreads.	[29]
Spreads	SLW (3–7%)	SFO	A formulation containing 4.29% wax and 24.13% water was suitable as a low-fat spread.	[107]
Margarine	MAG (1.77%) and CLW (2.66%)	HOSO	The oleogel margarine presented similar color as the commercial one. Oleogel was more resistant to temperature oscillations and an overall softer product. The consumers quickly detected the sensorial difference between the samples.	[108]
Margarine	BEW (10%)	SFO	Similar textural and rheological properties were obtained with 100% fat replaced compared to commercial control margarine. Formulated margarine presented lower content of SFC, and a higher melting point.	[109]

* CLW: candelilla wax; CRW: carnauba wax; BEW: bees wax; RBW: rice bran wax; SFW: sunflower wax; SLW: shellac wax; MAG: monoglycerides. ** SFO: sunflower oil; RBO: rice bran oil; OO: olive oil; SBO: soybean oil; RSO: rapeseed oil; HOSO: high oleic sunflower oil; LSO: linseed oil; HPO: hempseed oil; HZO: hazelnut oil.

**Table 5:** Application of oleogels in chocolate and confectionary products.

Application	Oleogelator	Liquid Phase	Major Outcomes	Reference
Chocolate	EC * (20 or 25%)	Ethanol	Adding EC to chocolate provided heat resistance. Oil migration at higher temperatures from the chocolate had been substantially reduced.	[41]
	MAG, EC, or BS:LE (10%)	CO **	MAG oleogel replaced 100% CB with a solid-like appearance. Oleogels performed soft texture and low SFC in chocolate. Thermal properties and polymorphic comparable to dark chocolate.	[110]
Chocolate paste	SLW (1.5%)	RSO	No oil loss from oleogel chocolate paste after four weeks of storage at 30 °C. The sample’s viscosity was comparable to the control sample’s. The yield strength was higher for the oleogel paste.	[29]
	MAG, BEW or PPW (5%)	PSO	Palm oil oleogels enhanced the mechanical parameters. MAG-containing samples showed a higher hardness. Oleogel palm oil mixture presented good oil binding capacity and potential fat replacement in chocolate spread.	[111]
	HPMC (1%) + XG (0.6%)	OO or SFO	100% fat replacement presented less homogeneous spreads. 50% replacement of oleogel presented sensory attributes similar to the control spread.	[112]
	CLW or MAG (10%)	WO	CLW led to the formation of a much firmer and spreadable product than MAG. CLW oleogel displayed an excellent tolerance to deformation. CLW-based oleogel spread exhibited good OBC (99.98%).	[113]
	MAG (20%)	CO	The reduced-fat chocolate spread displayed comparable sensory scores and acceptability to the control.	[114]
Filling cream	BEW (1.5–3.5%)	RBO	The SFC of the final filling products at body temperature was lower than 2.0% wt, suggesting a non-waxy mouthfeel. The melting enthalpy decreased with the increasing oleogel fraction.	[115]
Sandwich cookie filling	HPMC or MC (1%)	CNO	Great OBC with the replacement of shortening with oleogel. The partial replacements (50% and 75%) generated products similar to the commercial ones.	[9]
	MAG (10%)	HOSO	Decrease the OBC of fillings with the increase of oleogel content. Fillings formulated with 26% oleogel showed viscoelastic moduli values similar to the commercial filling cream used as a reference. A lower oil loss after 21 days of storage for the oleogel sample.	[116]
	CLW + MAG (10% total)	CNO	CLW:MAG 6:4 oleogel produced filling creams with textural properties similar to the shortening ones. The tomographic analysis and specific gravity showed that MAG oleogel samples presented a better-aerated structure.	[30]
	MAG + CLW + HF (5–10% total)	SBO or HOSO	Increased percentage of MAG or HF resulted in good aeration, greater hardness, and less oil loss compared with the standard with shortening.	[117]

* CLW: candelilla wax; CRW: carnauba wax; BEW: bees wax; RBW: rice bran wax; SFW: sunflower wax; SLW: shellac wax; MAG: monoglycerides; HPMC: hydroxypropyl methylcellulose. ** CNO: canola oil; SFO: sunflower oil; RBO: rice bran oil; OO: olive oil; SBO: soybean oil; RSO: rapeseed oil; HOSO: high oleic sunflower oil; CO: corn oil; WO: walnut oil; PSO: pomegranate seed oil.

**Table 6:** Application of oleogels in peanut butter.

**Peanut butter**	**Oleogelator**	**Liquid Phase**	**Outcomes**	**Reference**
	HPMC * or MC (2–10%)	PNO **	Reduced oil separation in peanut butter stabilized with freeze-dried HPMC and MC at levels as low as 0.2%. At levels of 2.2%, no oil loss was observed. Increased sample firmness and adhesiveness, which mimicked traditional peanut butter.	[120]
	BEW, CLW, RBW or SFW (1–2%)		Samples with 1.5–2.0% CLW or 1.0–1.5% RBW had fewer appearance and texture differences than the reference. Samples stabilized with 1.0–1.5% RBW scored closest to the commercial product.	[121]
	BEW, CLW, RBW or SFW (0.5–2%)		SFW oleogel had the highest firmness and the best OBC, the lowest oil separation after six months of storage at room temperature. >1.0% of all four waxes prevented long-term oil separation in peanut butter.	[119]
	RBW or CRW (1%)		RBW was the better stabilizer regardless of the mixing used. The spreadability, firmness, and oil binding capacity were close to the commercial peanut butter (natural and creamy).	[122]

* CLW: candelilla wax; CRW: carnauba wax; BEW: bees wax; RBW: rice bran wax; SFW: sunflower wax; HPMC: hydroxypropyl methylcellulose; MC: methylcellulose. ** PNO: peanut oil.

**Table 7:** Application of oleogels in frying medium.

**Frying medium**	**Oleogelator**	**Liquid Phase**	**Major Outcomes**	**Reference**
	CRW * (5 or 10%)	SBO **	The oleogels showed a faster change in viscosity with temperature. 16% less oil absorption than palm and soybean oil-fried noodles.	[123]
	CRW (5, 10 or 15%)	SBO	Mathri fried in oleogel retained more moisture, had better color and texture, and had a lower oil uptake than fried in soybean oil. Oleogel-fried mathri absorbed 27.7%, 22%, and 19.3% less oil.	[124]
	BEW (3 or 8%)	SFO	Oleogel-fried strips substantially absorbed less oil than the control sample. The samples cooked in oleogels were firmer, springier, and gummier than the control sample. Oleogel-fried potatoes had better sensory ratings, according to sensory analysis.	[125]

* CRW: carnauba wax; BEW: bees wax. ** SBO: soybean oil; SFO: sunflower oil.

## References

[1] Żarnowski A., Jankowski M., Gujski M. (2022). Public Awareness of Diet-Related Diseases and Dietary Risk Factors: A 2022 Nationwide Cross-Sectional Survey among Adults in Poland. Nutrients.

[2] Roche H.M. (2005). Fatty acids and the metabolic syndrome. Proc. Nutr. Soc..

[3] WHO (2018). WHO Plan to Eliminate Industrially-Produced Trans-Fatty Acids from Global Food Supply. https://www.who.int/news/item/14-05-2018-who-plan-to-eliminate-industrially-produced-trans-fatty-acids-from-global-food-supply.

[4] Berry S.E., Bruce J.H., Steenson S., Stanner S., Buttriss J.L., Spiro A., Gibson P.S., Bowler I., Dionisi F., Farrell L. (2019). Interesterified fats: What are they and why are they used? A briefing report from the Roundtable on Interesterified Fats in Foods. Nutr. Bull..

[5] Menaa F., Menaa A., Tréton J., Menaa B. (2013). Technological approaches to minimize industrial trans fatty acids in foods. J. Food Sci..

[6] Colla K., Costanzo A., Gamlath S. (2018). Fat replacers in baked food products. Foods.

[7] Banaś K., Harasym J. (2021). Natural gums as oleogelators. Int. J. Mol. Sci..

[8] Cui X., Saleh A.S.M., Yang S., Wang N., Wang P., Zhu M., Xiao Z. (2022). Oleogels as Animal Fat and Shortening Replacers: Research Advances and Application Challenges. Food Rev. Int..

[9] Tanti R., Barbut S., Marangoni A.G. (2016). Hydroxypropyl methylcellulose and methylcellulose structured oil as a replacement for shortening in sandwich cookie creams. Food Hydrocoll..

[10] Malvano F., Laudisio M., Albanese D., d’Amore M., Marra F. (2022). Olive Oil-Based Oleogel as Fat Replacer in a Sponge Cake: A Comparative Study and Optimization. Foods.

[11] Botega D.C.Z., Marangoni A.G., Smith A.K., Goff H.D. (2013). The Potential Application of Rice Bran Wax Oleogel to Replace Solid Fat and Enhance Unsaturated Fat Content in Ice Cream. J. Food Sci..

[12] Flöter E., Wettlaufer T., Conty V., Scharfe M. (2021). Oleogels—Their applicability and methods of characterization. Molecules.

[13] Lim J., Hwang H.S., Lee S. (2017). Oil-structuring characterization of natural waxes in canola oil oleogels: Rheological, thermal, and oxidative properties. Appl. Biol. Chem..

[14] da Pieve S., Calligaris S., Co E., Nicoli M.C., Marangoni A.G. (2010). Shear Nanostructuring of monoglyceride organogels. Food Biophys..

[15] Bot A., Veldhuizen Y.S.J., den Adel R., Roijers E.C. (2009). Non-TAG structuring of edible oils and emulsions. Food Hydrocoll..

[16] Si H., Cheong L.Z., Huang J., Wang X. (2016). Physical Properties of Soybean Oleogels and Oil Migration Evaluation in Model Praline System. J. Am. Oil Chem. Soc..

[17] Zetzl A.K., Gravelle A.J., Kurylowicz M., Dutcher J., Barbut S., Marangoni A.G. (2014). Microstructure of ethylcellulose oleogels and its relationship to mechanical properties. Food Struct..

[18] Totosaus A., Gonzaléz-Gonzaléz R., Fragoso M. (2016). Influence of the type of cellulosic derivatives on the texture, and oxidative and thermal stability of soybean oil oleogel. Grasas. Y Aceites.

[19] da Silva T.L.T., Arellano D.B., Martini S. (2018). Physical Properties of Candelilla Wax, Monoacylglycerols, and Fully Hydrogenated Oil Oleogels. J. Am. Oil Chem. Soc..

[20] Okuro P.K., Malfatti-Gasperini A.A., Vicente A.A., Cunha R.L. (2018). Lecithin and phytosterols-based mixtures as hybrid structuring agents in different organic phases. Food Res. Int..

[21] de Godoi K.R.R., Basso R.C., Ming C.C., da Silva V.M., da Cunha R.L., Barrera-Arellano D., Ribeiro A.P.B. (2019). Physicochemical and rheological properties of soybean organogels: Interactions between different structuring agents. Food Res. Int..

[22] da Silva T.L.T., Fernandes G.D., Arellano D.B. (2022). The combination of monoglycerides, wax and hardfat on oleogels structuration. Braz. J. Food Technol..

[23] Teodoro da Silva T.L., Danthine S. (2022). Influence of sonocrystallization on lipid crystals multicomponent oleogels structuration and physical properties. Food Res. Int..

[24] Patel A.R., Dewettinck K. (2015). Comparative evaluation of structured oil systems: Shellac oleogel, HPMC oleogel, and HIPE gel. Eur. J. Lipid. Sci. Technol..

[25] Pang M., Kang S., Liu L., Ma T., Zheng Z., Cao L. (2023). Physicochemical Properties and Cookie-Making Performance as Fat Replacer of Wax-Based Rice Bran Oil Oleogels. Gels.

[26] Giacomozzi A.S., Carrín M.E., Palla C.A. (2018). Muffins Elaborated with Optimized Monoglycerides Oleogels: From Solid Fat Replacer Obtention to Product Quality Evaluation. J. Food Sci..

[27] Ferro A.C., de Souza Paglarini C., Rodrigues Pollonio M.A., Lopes Cunha R. (2021). Glyceryl monostearate-based oleogels as a new fat substitute in meat emulsion. Meat Sci..

[28] Barbut S., Wood J., Marangoni A. (2016). Potential use of organogels to replace animal fat in comminuted meat products. Meat Sci..

[29] Patel A.R., Rajarethinem P., Grędowska A., Turhan O., Lesaffer A., De Vos W.H., Van de Walle D., Dewettinck K. (2014). Edible Applications of Shellac Oleogels: Spreads, Chocolate Paste and Cakes. Food Funct..

[30] Kim M., Hwang H.S., Jeong S., Lee S. (2022). Utilization of oleogels with binary oleogelator blends for filling creams low in saturated fat. LWT Food Sci. Technol..

[31] Hwang H.S., Winkler-Moser J.K. (2020). Properties of margarines prepared from soybean oil oleogels with mixtures of candelilla wax and beeswax. J. Food Sci..

[32] Ögutcu M., Yilmaz E. (2014). Oleogels of virgin olive oil with carnauba wax and monoglyceride as spreadable products. Grasas Y Aceites.

[33] Roy S., Hussain S.A., Prasad W.G., Khetra Y. (2022). Quality attributes of high protein ice cream prepared by incorporation of whey protein isolate. Appl. Food Res..

[34] Puscas A., Muresan V., Socaciu C., Muste S. (2020). Oleogels in food: A review of current and potential applications. Foods.

[35] Devi A., Khatkar B.S. (2016). Physicochemical, rheological and functional properties of fats and oils in relation to cookie quality: A review. J. Food Sci. Technol..

[36] Doan C.D., Tavernier I., Danthine S., Rimaux T., Dewettinck K. (2018). Physical compatibility between wax esters and triglycerides in hybrid shortenings and margarines prepared in rice bran oil. J. Sci. Food Agric..

[37] Rios R.V., Pessanha M.D.F., de Almeida P.F., Viana C.L., da Silva Lannes S.C. (2014). Application of fats in some food products. Food Sci. Technol..

[38] Demirkesen I., Mert B. (2019). Utilization of Beeswax Oleogel-Shortening Mixtures in Gluten-Free Bakery Products. J. Am. Oil Chem. Soc..

[39] Bemer H.L., Limbaugh M., Cramer E.D., Harper W.J., Maleky F. (2016). Vegetable organogels incorporation in cream cheese products. Food Res. Int..

[40] Barbut S., Wood J., Marangoni A.G. (2016). Effects of Organogel Hardness and Formulation on Acceptance of Frankfurters. J. Food Sci..

[41] Stortz T.A., Marangoni A.G. (2013). Ethylcellulose solvent substitution method of preparing heat resistant chocolate. Food Res. Int..

[42] Temkov M., Mureșan V. (2021). Tailoring the structure of lipids, oleogels and fat replacers by different approaches for solving the trans-fat issue—A review. Foods.

[43] Wettlaufer T., Flöter E. (2022). Wax based oleogels and their application in sponge cakes. Food Funct..

[44] Siraj N., Shabbir M.A., Ahmad T., Sajjad A., Khan M.R., Khan M.I., Butt M.S. (2015). Organogelators as a Saturated Fat Replacer for Structuring Edible Oils. Int. J. Food Prop..

[45] Dassanayake L.S.K., Kodali D.R., Ueno S. (2011). Formation of oleogels based on edible lipid materials. Curr. Opin. Colloid. Interface Sci..

[46] Patel A.R. (2017). A colloidal gel perspective for understanding oleogelation. Curr. Opin. Food Sci..

[47] Manzoor S., Masoodi F.A., Naqash F., Rashid R. (2022). Oleogels: Promising alternatives to solid fats for food applications. Food Hydrocoll. Health.

[48] Podmaniczky F., Gránásy L. (2022). Molecular scale hydrodynamic theory of crystal nucleation and polycrystalline growth. J. Cryst. Growth.

[49] Pehlivanoğlu H., Demirci M., Toker O.S., Konar N., Karasu S., Sagdic O. (2018). Oleogels, a promising structured oil for decreasing saturated fatty acid concentrations: Production and food-based applications. Crit. Rev. Food Sci. Nutr..

[50] Ribeiro A.P.B., Masuchi M.H., Miyasaki E.K., Domingues M.A.F., Stroppa V.L.Z., De Oliveira G.M., Kieckbusch T.G. (2015). Crystallization modifiers in lipid systems. J. Food Sci. Technol..

[51] Álvarez M.D., Cofrades S., Pérez-Mateos M., Saiz A., Herranz B. (2022). Development and Physico-Chemical Characterization of Healthy Puff Pastry Margarines Made from Olive-Pomace Oil. Foods.

[52] Sarkisyan V., Sobolev R., Frolova Y., Malinkin A., Makarenko M., Kochetkova A. (2021). Beeswax Fractions Used as Potential Oil Gelling Agents. J. Am. Oil Chem. Soc..

[53] Gravelle A.J., Marangoni A.G. (2021). Dataset on the small- and large deformation mechanical properties of emulsion-filled gelatin hydrogels as a model particle-filled composite food gel. Data Br..

[54] Co E.D., Marangoni A.G. (2012). Organogels: An alternative edible oil-structuring method. J. Am. Oil Chem. Soc..

[55] Wang Q., Espert M., Larrea V., Quiles A., Salvador A., Sanz T. (2023). Comparison of different indirect approaches to design edible oleogels based on cellulose ethers. Food Hydrocoll..

[56] Patel A.R., Mankoč B., Bin Sintang M.D., Lesaffer A., Dewettinck K. (2015). Fumed silica-based organogels and ‘aqueous-organic’ bigels. R Soc. Chem..

[57] Whitby C.P. (2020). Structuring Edible Oils With Fumed Silica Particles. Front. Sustain. Food Syst..

[58] Zhao M., Lan Y., Cui L., Monono E., Rao J., Chen B. (2020). Formation, characterization, and potential food application of rice bran wax oleogels: Expeller-pressed corn germ oil versus refined corn oil. Food Chem..

[59] Davidovich-Pinhas M., Barbut S., Marangoni A.G. (2015). The role of surfactants on ethylcellulose oleogel structure and mechanical properties. Carbohydr. Polym..

[60] Martins A.J., Vicente A.A., Pastrana L.M., Cerqueira M.A. (2020). Oleogels for development of health-promoting food products. Food Sci. Hum. Wellness.

[61] Tan S., Peh E.W.-Y., Marangoni A.G., Henry C.J. (2017). Effects of liquid oil vs. oleogel co-ingested with a carbohydrate-rich meal on human blood triglycerides, glucose, insulin and appetite. Food Funct..

[62] Limpimwong W., Kumrungsee T., Kato N., Yanaka N., Thongngam M. (2017). Rice bran wax oleogel: A potential margarine replacement and its digestibility effect in rats fed a high-fat diet. J. Funct. Foods.

[63] Dong L., Lv M., Gao X., Zhang L., Rogers M., Cao Y., Lan Y. (2020). In vitro gastrointestinal digestibility of phytosterol oleogels: Influence of self-assembled microstructures on emulsification efficiency and lipase activity. Food Funct..

[64] Patel A.R., Nicholson R.A., Marangoni A.G. (2020). Applications of fat mimetics for the replacement of saturated and hydrogenated fat in food products. Curr. Opin. Food Sci..

[65] Patel A.R., Cludts N., Bin Sintang M.D., Lewille B., Lesaffer A., Dewettinck K. (2014). Polysaccharide-based oleogels prepared with an emulsion-templated approach. ChemPhysChem.

[66] Jang A., Bae W., Hwang H.S., Lee H.G., Lee S. (2015). Evaluation of canola oil oleogels with candelilla wax as an alternative to shortening in baked goods. Food Chem..

[67] Mert B., Demirkesen I. (2016). Reducing saturated fat with oleogel/shortening blends in a baked product. Food Chem..

[68] Mert B., Demirkesen I. (2016). Evaluation of highly unsaturated oleogels as shortening replacer in a short dough product. LWT Food Sci. Technol..

[69] Hwang H.S., Singh M., Lee S. (2016). Properties of Cookies Made with Natural Wax-Vegetable Oil Organogels. J. Food Sci..

[70] Li S., Zhu L., Li X., Wu G., Liu T., Qi X., Jin Q., Wang X., Zhang H. (2022). Determination of characteristic evaluation indexes for novel cookies prepared with wax oleogels. J. Sci. Food Agric..

[71] Li S., Wu G., Li X., Jin Q., Wang X., Zhang H. (2021). Roles of gelator type and gelation technology on texture and sensory properties of cookies prepared with oleogels. Food Chem..

[72] Li S., Zhu L., Wu G., Jin Q., Wang X., Zhang H. (2022). Relationship between the microstructure and physical properties of emulsifier based oleogels and cookies quality. Food Chem..

[73] Schubert M., Erlenbusch N., Wittland S., Nikolay S., Hetzer B., Matthäus B. (2022). Rapeseed Oil Based Oleogels for the Improvement of the Fatty Acid Profile Using Cookies as an Example. Eur. J. Lipid Sci. Technol..

[74] Zhao M., Lan Y., Cui L., Monono E., Rao J., Chen B. (2020). Physical properties and cookie-making performance of oleogels prepared with crude and refined soybean oil: A comparative study. Food Funct..

[75] Jiang Q., Yu Z., Meng Z. (2022). Double network oleogels co-stabilized by hydroxypropyl methylcellulose and monoglyceride crystals: Baking applications. Int. J. Biol. Macromol..

[76] Oh I.K., Lee S. (2018). Utilization of foam structured hydroxypropyl methylcellulose for oleogels and their application as a solid fat replacer in muffins. Food Hydrocoll..

[77] Giacomozzi A.S., Carrín M.E., Palla C.A. (2022). Muffins made with monoglyceride oleogels: Impact of fat replacement on sensory properties and fatty acid profile. J. Am. Oil Chem. Soc..

[78] Alvarez-Ramirez J., Vernon-Carter E.J., Carrera-Tarela Y., Garcia A., Roldan-Cruz C. (2020). Effects of candelilla wax/canola oil oleogel on the rheology, texture, thermal properties and in vitro starch digestibility of wheat sponge cake bread. LWT Food Sci. Technol..

[79] Zhao M., Rao J., Chen B. (2022). Effect of high oleic soybean oil oleogels on the properties of doughs and corresponding bakery products. J. Am. Oil Chem. Soc..

[80] Tanislav A.E., Pușcaș A., Păucean A., Mureșan A.E., Semeniuc C.A., Mureșan V., Mudura E. (2022). Evaluation of Structural Behavior in the Process Dynamics of Oleogel-Based Tender Dough Products. Gels.

[81] da Silva T.L.T., Arellano D.B., Martini S. (2019). Effect of Water Addition on Physical Properties of Emulsion Gels. Food Biophys.

[82] Kim J.Y., Lim J., Lee J., Hwang H., Lee S. (2017). Utilization of Oleogels as a Replacement for Solid Fat in Aerated Baked Goods: Physicochemical, Rheological, and Tomographic Characterization. J. Food Sci..

[83] Botega D.C.Z., Marangoni A.G., Smith A.K., Goff H.D. (2013). Development of formulations and processes to incorporate wax oleogels in ice cream. J. Food Sci..

[84] Airoldi R., Ract J.N.R. (2022). Potential Use of Carnauba Wax Oleogel to Replace Saturated Fat in Ice Cream. J. Am. Oil Chem. Soc..

[85] Jing X., Chen Z., Tang Z., Tao Y., Huang Q., Wu Y., Zhang H., Li X., Liang J., Liu Z. (2022). Preparation of camellia oil oleogel and its application in an ice cream system. LWT Food Sci. Technol..

[86] Moriano M.E., Alamprese C. (2017). Organogels as novel ingredients for low saturated fat ice creams. LWT Food Sci. Technol..

[87] Park C., Bemer H.L., Maleky F. (2018). Oxidative Stability of Rice Bran Wax Oleogels and an Oleogel Cream Cheese Product. J. Am. Oil Chem. Soc..

[88] Huang H., Hallinan R., Maleky F. (2018). Comparison of different oleogels in processed cheese products formulation. Int. J. Food Sci. Technol..

[89] Moon K., Choi K.O., Jeong S., Kim Y.W., Lee S. (2021). Solid fat replacement with canola oil-carnauba wax oleogels for dairy-free imitation cheese low in saturated fat. Foods.

[90] Gab-Allah R. (2018). Manufacture of pickled and un-pickled high fat soft cheese using olive and sunflower oleogels. Sciences.

[91] Brighenti M., Govindasamy-Lucey S., Lim K., Nelson K., Lucey J.A. (2008). Characterization of the rheological, textural, and sensory properties of samples of commercial US cream cheese with different fat contents. J. Dairy Sci..

[92] Gómez-Estaca J., Pintado T., Jiménez-Colmenero F., Cofrades S. (2019). Assessment of a healthy oil combination structured in ethyl cellulose and beeswax oleogels as animal fat replacers in low-fat, PUFA-enriched pork burgers. Food Bioprocess Technol..

[93] Adili L., Roufegarinejad L., Tabibiazar M., Hamishehkar H., Alizadeh A. (2020). Development and characterization of reinforced ethyl cellulose based oleogel with adipic acid: Its application in cake and beef burger. LWT Food Sci. Technol..

[94] Martins A.J., Lorenzo J.M., Franco D., Vicente A.A., Cunha R.L., Pastrana L.M., Quiñones J., Cerqueira M.A. (2019). Omega-3 and Polyunsaturated Fatty Acids-Enriched Hamburgers Using Sterol-Based Oleogels. Eur. J. Lipid Sci. Technol..

[95] Oh I., Lee J.H., Lee H.G., Lee S. (2019). Feasibility of hydroxypropyl methylcellulose oleogel as an animal fat replacer for meat patties. Food Res. Int..

[96] Moghtadaei M., Soltanizadeh N., Goli S.A.H. (2018). Production of sesame oil oleogels based on beeswax and application as partial substitutes of animal fat in beef burger. Food Res. Int..

[97] Panagiotopoulou E., Moschakis T., Katsanidis E. (2016). Sunflower oil organogels and organogel-in-water emulsions (part II): Implementation in frankfurter sausages. LWT Food Sci. Technol..

[98] Wolfer T.L., Acevedo N.C., Prusa K.J., Sebranek J.G., Tarté R. (2018). Replacement of pork fat in frankfurter-type sausages by soybean oil oleogels structured with rice bran wax. Meat Sci..

[99] Tarté R., Paulus J.S., Acevedo N.C., Prusa K.J., Lee S.L. (2020). High-oleic and conventional soybean oil oleogels structured with rice bran wax as alternatives to pork fat in mechanically separated chicken-based bologna sausage. Lwt.

[100] Zampouni K., Soniadis A., Dimakopoulou-Papazoglou D., Moschakis T., Biliaderis C.G., Katsanidis E. (2022). Modified fermented sausages with olive oil oleogel and NaCl–KCl substitution for improved nutritional quality. Lwt.

[101] Pintado T., Cofrades S. (2020). Quality characteristics of healthy dry fermented sausages formulated with a mixture of olive and chia oil structured in oleogel or emulsion gel as animal fat replacer. Foods.

[102] Hwang H.S., Singh M., Bakota E.L., Winkler-Moser J.K., Kim S., Liu S.X. (2013). Margarine from organogels of plant wax and soybean oil. J. Am. Oil Chem. Soc..

[103] Hwang H.S., Singh M., Winkler-Moser J.K., Bakota E.L., Liu S.X. (2014). Preparation of margarines from organogels of sunflower wax and vegetable oils. J. Food Sci..

[104] Hwang H.S., Kim S., Winkler-Moser J.K., Lee S., Liu S.X. (2022). Feasibility of hemp seed oil oleogels structured with natural wax as solid fat replacement in margarine. J. Am. Oil Chem. Soc..

[105] Chai X., Zhang Y., Shi Y., Liu Y. (2022). Crystallization and Structural Properties of Oleogel-Based Margarine. Molecules.

[106] Yilmaz E., Ogutcu M. (2015). Oleogels as spreadable fat and butter alternatives: Sensory description and consumer perception. R Soc. Chem..

[107] Puscas A., Muresan V. (2022). The Feasibility of Shellac Wax Emulsion Oleogels as Low-Fat Spreads Analyzed by Means of Multidimensional Statistical Analysis. Gels.

[108] da Silva T.L.T., Chaves K.F., Fernandes G.D., Rodrigues J.B., Bolini H.M.A., Arellano D.B. (2018). Sensory and Technological Evaluation of Margarines With Reduced Saturated Fatty Acid Contents Using Oleogel Technology. J. Am. Oil Chem. Soc..

[109] Abdolmaleki K., Alizadeh L., Nayebzadeh K., Baranowska H.M., Kowalczewski P.Ł., Khaneghah A.M. (2022). Potential Application of Hydrocolloid-Based Oleogel and Beeswax Oleogel as Partial Substitutes of Solid Fat in Margarine. Appl. Sci..

[110] Li L., Liu G. (2019). Corn oil-based oleogels with different gelation mechanisms as novel cocoa butter alternatives in dark chocolate. J. Food Eng..

[111] Fayaz G., Goli S.A.H., Kadivar M., Valoppi F., Barba L., Calligaris S., Nicoli M.C. (2017). Potential application of pomegranate seed oil oleogels based on monoglycerides, beeswax and propolis wax as partial substitutes of palm oil in functional chocolate spread. LWT Food Sci. Technol..

[112] Bascuas S., Espert M., Llorca E., Quiles A., Salvador A., Hernando I. (2021). Structural and sensory studies on chocolate spreads with hydrocolloid-based oleogels as a fat alternative. LWT Food Sci. Technol..

[113] Pușcaș A., Tanislav A.E., Mureșan A.E., Fărcaș A.C., Mureșan V. (2022). Walnut Oil Oleogels as Milk Fat Replacing System for Commercially Available Chocolate Butter. Gels.

[114] Tirgarian B., Yadegari H., Bagheri A., Neshagaran E., Mardani M., Farmani J. (2023). Reduced-fat chocolate spreads developed by water-in-oleogel emulsions. J. Food Eng..

[115] Doan C.D., Patel A.R., Tavernier I., de Clercq N., van Raemdonck K. (2016). The feasibility of wax-based oleogel as a potential co-structurant with palm oil in low-saturated fat confectionery fillings. Eur. J. Lipid Sci. Technol..

[116] Palla C.A., Wasinger M.F., Carrín M.E. (2021). Monoglyceride oleogels as fat replacers in filling creams for sandwich cookies. J. Sci. Food Agric..

[117] da Silva T.L.T., Fernandes G.D., Arellano D.B. (2021). Development of reduced saturated fat cookie fillings using multicomponent oleogels. J. Am. Oil Chem. Soc..

[118] Stortz T.A., Marangoni A.G. (2011). Heat resistant chocolate. Trends Food Sci. Technol..

[119] Winkler-Moser J.K., Anderson J., Byars J.A., Singh M., Hwang H.S. (2019). Evaluation of Beeswax, Candelilla Wax, Rice Bran Wax, and Sunflower Wax as Alternative Stabilizers for Peanut Butter. J. Am. Oil Chem. Soc..

[120] Tanti R., Barbut S., Marangoni A.G. (2016). Food Hydrocolloids Oil stabilization of natural peanut butter using food grade polymers. Food Hydrocoll..

[121] Winkler-Moser J.K., Anderson J.A., Hwang H.S. (2022). Texture and flavor evaluation of peanut butter stabilized with natural waxes. J. Food Sci..

[122] Ferdaus M.J., Blount R.J.S., da Silva R.C. (2022). Assessment of Natural Waxes as Stabilizers in Peanut Butter. Foods.

[123] Lim J., Jeong S., Oh I.K., Lee S. (2017). Evaluation of soybean oil-carnauba wax oleogels as an alternative to high saturated fat frying media for instant fried noodles. LWT Food Sci. Technol..

[124] Chauhan D.S., Khare A., Lal A.B., Bebartta R.P. (2022). Utilising oleogel as a frying medium for deep fried Indian traditional product (Mathri) to reduce oil uptake. J. Indian Chem. Soc..

[125] Aydeniz Guneser B., Yılmaz E., Uslu E.K. (2021). Sunflower Oil–Beeswax Oleogels Are Promising Frying Medium for Potato Strips. Eur. J. Lipid Sci. Technol..

